# Effect of ready-to-use supplementary food on mortality in severely immunocompromised HIV-infected individuals in Africa initiating antiretroviral therapy (REALITY): an open-label, parallel-group, randomised controlled trial

**DOI:** 10.1016/S2352-3018(18)30038-9

**Published:** 2018-04-10

**Authors:** Jane Mallewa, Alexander J Szubert, Peter Mugyenyi, Ennie Chidziva, Margaret J Thomason, Priscilla Chepkorir, George Abongomera, Keith Baleeta, Anthony Etyang, Colin Warambwa, Betty Melly, Shepherd Mudzingwa, Christine Kelly, Clara Agutu, Helen Wilkes, Sanele Nkomani, Victor Musiime, Abbas Lugemwa, Sarah L Pett, Mutsa Bwakura-Dangarembizi, Andrew J Prendergast, Diana M Gibb, A Sarah Walker, James A Berkley, REALITY trial team, Peter Mugyenyi, Cissy Kityo, Victor Musiime, Priscilla Wavamunno, Esther Nambi, Paul Ocitti, Milly Ndigendawani, Sheila Kabahenda, Mable Kemigisa, Juliet Acen, David Francis Olebo, Gordon Mpamize, Alex Amone, David Okweny, Andrew Mbonye, Florence Nambaziira, Angela Rweyora, Mary Kangah, Beatrice Kabaswahili, James Abach, George Abongomera, Joseph Omongin, Irene Aciro, Aleti Philliam, Beatrice Arach, Emmanuel Ocung, Geoffrey Amone, Peter Miles, Claudia Adong, Constance Tumsuiime, Patrick Kidega, Ben Otto, Florence Apio, Keith Baleeta, Andrew Mukuye, Mary Abwola, Fred Ssennono, David Baliruno, Stephen Tuhirwe, Ronald Namisi, Fredrick Kigongo, Dickson Kikyonkyo, Furaha Mushahara, David Okweny, Julian Tusiime, Alex Musiime, Agnes Nankya, Dickens Atwongyeire, Sowal Sirikye, Sula Myalo, Nelson Noowe, Abbas Lugemwa, Mariam Kasozi, Sandra Mwebe, Lorna Atwine, Tapson Senkindu, Ian Natuhurira, Chrispus Katemba, Emily Ninsiima, Moses Acaku, Joy Kyomuhangi, Rogers Ankunda, Deogratious Tukwasibwe, Lillian Ayesiga, James Hakim, Kusum Nathoo, Mutsa Bwakura-Dangarembizi, Andrew Reid, Ennie Chidziva, Tawand Mhute, Gloria Tinago, Joyline Bhiri, Shepherd Mudzingwa, Misheck Phiri, John Steamer, Ruth Nhema, Colin Warambwa, Godfrey Musoro, Shirley Mutsai, Beauty Nemasango, Columbus Moyo, Stuart Chitongo, Kennias Rashirai, Sydney Vhembo, Brian Mlambo, Sanele Nkomani, Buxton Ndemera, Marko Willard, Chipo Berejena, Yeukai Musodza, Patience Matiza, Boniface Mudenge, Vongai Guti, Anthony Etyang, Clara Agutu, Jay Berkley, Kathryn Maitland, Patricia Njuguna, Shalton Mwaringa, Timothy Etyang, Ken Awuondo, Stephen Wale, Jimmy Shangala, Jefwa Kithunga, Salim Mwarumba, Salma Said Maitha, Robert Mutai, Margaret Lozi Lewa, Gabriel Mwambingu, Alfred Mwanzu, Connie Kalama, Helen Latham, Joyce Shikuku, Amos Fondo, Anne Njogu, Connie Khadenge, Bryan Mwakisha, Abraham Siika, Kara Wools-Kaloustian, Winston Nyandiko, Priscilla Chepkorir-Cheruiyot, Allan Sudoi, Simon Wachira, Betty Meli, Mercy Karoney, Agnes Nzioka, Michael Tanui, Martha Mokaya, Wilson Ekiru, Chris Mboya, Dorothy Mwimali, Cecilia Mengich, Julie Choge, Wilfred Injera, Kennedy Njenga, Salinah Cherutich, Millicent Anyango Orido, Gerald Omondi Lwande, Peter Rutto, Alice Mudogo, Irene Kutto, Amina Shali, Linda Jaika, Hellen Jerotich, Mowlem Pierre, Jane Mallewa, Symon Kaunda, Joep Van Oosterhout, Bernadette O'Hare, Robert Heydermann, Carmen Gonzalez, Nettie Dzabala, Christine Kelly, Brigitte Denis, George Selemani, Linda Nyondo- Mipando, Emmie Chirwa, Peter Banda, Linley Mvula, Harrison Msuku, Milton Ziwoya, Yollam Manda, Simon Nicholas, Clemens Masesa, Thandi Mwalukomo, Lumbani Makhaza, Irene Sheha, Joseph Bwanali, Molly Limbuni, Diana M Gibb, Margaret J Thomason, Ann Sarah Walker, Sarah L Pett, Alexander J Szubert, Anna Griffiths, Helen Wilkes, Chathurika Rajapakse, Moira J Spyer, Andrew J Prendergast, Nigel Klein, Mary Rauchenberger, Nadine Van Looy, Emma Little, Keith Fairbrother, Frances Cowan, Janet Seeley, Sarah Bernays, Rachel Kawuma, Zivai Mupambireyi

**Affiliations:** a Department/College of Medicine and Malawi-Liverpool-Wellcome Trust Clinical Research Programme, Blantyre, Malawi; b Medical Research Council Clinical Trials Unit at University College London, London, UK; c The Kirby Institute, University of New South Wales, Sydney, Australia; d Joint Clinical Research Centre, Kampala, Uganda; e University of Zimbabwe Clinical Research Centre, Harare, Zimbabwe; f Moi University School of Medicine, Eldoret, Kenya; g Joint Clinical Research Centre, Gulu, Uganda; h Joint Clinical Research Centre, Mbale, Uganda; i KEMRI/Wellcome Trust Research Programme, Kilifi, Kenya; j Joint Clinical Research Centre, Mbarara, Uganda; k Queen Mary University of London, London, UK

## Abstract

**Background:**

In sub-Saharan Africa, severely immunocompromised HIV-infected individuals have a high risk of mortality during the first few months after starting antiretroviral therapy (ART). We hypothesise that universally providing ready-to-use supplementary food (RUSF) would increase early weight gain, thereby reducing early mortality compared with current guidelines recommending ready-to-use therapeutic food (RUTF) for severely malnourished individuals only.

**Methods:**

We did a 2 × 2 × 2 factorial, open-label, parallel-group trial at inpatient and outpatient facilities in eight urban or periurban regional hospitals in Kenya, Malawi, Uganda, and Zimbabwe. Eligible participants were ART-naive adults and children aged at least 5 years with confirmed HIV infection and a CD4 cell count of fewer than 100 cells per μL, who were initiating ART at the facilities. We randomly assigned participants (1:1) to initiate ART either with (RUSF) or without (no-RUSF) 12 weeks' of peanut-based RUSF containing 1000 kcal per day and micronutrients, given as two 92 g packets per day for adults and one packet (500 kcal per day) for children aged 5–12 years, regardless of nutritional status. In both groups, individuals received supplementation with RUTF only when severely malnourished (ie, body-mass index [BMI] <16–18 kg/m^2^ or BMI-for-age *Z* scores <–3 for children). We did the randomisation with computer-generated, sequentially numbered tables with different block sizes incorporated within an online database. Randomisation was stratified by centre, age, and two other factorial randomisations, to 12 week adjunctive raltegravir and enhanced anti-infection prophylaxis (reported elsewhere). Clinic visits were scheduled at weeks 2, 4, 8, 12, 18, 24, 36, and 48, and included nurse assessment of vital status and symptoms and dispensing of all medication including ART and RUSF. The primary outcome was mortality at week 24, analysed by intention to treat. Secondary outcomes included absolute changes in weight, BMI, and mid-upper-arm circumference (MUAC). Safety was analysed in all randomly assigned participants. Follow-up was 48 weeks. This trial is registered with ClinicalTrials.gov (NCT01825031) and the ISRCTN registry (43622374).

**Findings:**

Between June 18, 2013, and April 10, 2015, we randomly assigned 1805 participants to treatment: 897 to RUSF and 908 to no-RUSF. 56 (3%) were lost-to-follow-up. 96 (10·9%, 95% CI 9·0–13·1) participants allocated to RUSF and 92 (10·3%, 8·5–12·5) to no-RUSF died within 24 weeks (hazard ratio 1·05, 95% CI 0·79–1·40; log-rank p=0·75), with no evidence of interaction with the other randomisations (both p>0·7). Through 48 weeks, adults and adolescents aged 13 years and older in the RUSF group had significantly greater gains in weight, BMI, and MUAC than the no-RUSF group (p=0·004, 0·004, and 0·03, respectively). The most common type of serious adverse event was specific infections, occurring in 90 (10%) of 897 participants assigned RUSF and 87 (10%) of 908 assigned no-RUSF. By week 48, 205 participants had serious adverse events in both groups (p=0·81), and 181 had grade 4 adverse events in the RUSF group compared with 172 in the non-RUSF group (p=0·45).

**Interpretation:**

In severely immunocompromised HIV-infected individuals, providing RUSF universally at ART initiation, compared with providing RUTF to severely malnourished individuals only, improved short-term weight gain but not mortality. A change in policy to provide nutritional supplementation to all severely immunocompromised HIV-infected individuals starting ART is therefore not warranted at present.

**Funding:**

Joint Global Health Trials Scheme (UK Medical Research Council, UK Department for International Development, and Wellcome Trust).

**Research in context**

**Evidence before this study**

We searched published articles in PubMed without date or language restriction containing the terms (“HIV”[MeSH Terms] OR “HIV”[All Fields]) AND ((OR (“nutritional”[All Fields]) OR “nutritional status”[All Fields] OR “nutrition”[All Fields] OR nutritional[All Fields] OR feeding[All Fields]) AND ((“dietary supplements”[MeSH Terms] OR (“dietary”[All Fields] AND “supplements”[All Fields]) OR “dietary supplements”[All Fields] OR “supplement”[All Fields]) OR “supplementation”[All Fields])) AND (“clinical trials as topic”[MeSH Terms] OR “trial”[All Fields]). Previous studies have included severely malnourished and non-severely malnourished HIV-infected individuals, predominantly in the absence of severe immunosuppression. The findings showed that lipid-based nutritional supplementation at ART initiation was associated with weight gain, but provided no evidence of a reduction in mortality. A systematic review concluded that the effect of nutritional supplementation on mortality, morbidity, and disease outcomes was uncertain. No nutritional trials reported targeting individuals who were at high risk of mortality after starting ART because of severe immunosuppression.

**Added value of this study**

In our randomised controlled trial, the group universally receiving RUSF gained more weight in the short term than the group who received supplementation only if they were severely malnourished according to current guidelines. However, there was no significant difference in deaths, adverse events, viral load suppression, or CD4 cell counts.

**Implications of all the available evidence**

HIV-infected individuals with severe malnutrition should continue to be identified and treated, but a change in policy to provide lipid-based nutritional supplements universally to all severely immunocompromised HIV-infected individuals starting ART is not warranted.

## Introduction

WHO guidelines recommend universal antiretroviral therapy (ART) for HIV-infected individuals regardless of their CD4 cell counts.^1^ However, 20–25% of HIV-infected individuals in sub-Saharan Africa still present for care with severe immunosuppression (ie, CD4 cell count <100 cells per μL),^2^ of whom around 10% die within 3 months of ART initiation.^3^ Early mortality is higher in adults and children with low CD4 cell counts or low body-mass index (BMI), even if these levels are above those indicating severe malnutrition.^4^

Macronutrient and micronutrient undernutrition is common in HIV-infected individuals and might be a result of anorexia, swallowing difficulties, food insecurity, frequent illness, intestinal dysfunction, and the increased nutrient demands posed by immune activation and opportunistic infections.^5^ Food insecurity has also been associated with poor recovery of CD4 cell counts, reduced adherence to ART, and low retention in care.^5^, ^6^ Energy requirements are increased by about 10% in HIV-infected individuals who are not sick, and by 20–50% during symptomatic disease and initial recovery.^7^ Many patients report acute hunger after starting ART, but might be unable to increase their calorie intake.^8^ However, precise macronutrient and micronutrient requirements for individuals initiating ART at different stages of HIV disease have not been defined.

WHO and UNAIDS recommendations for people living with HIV focus on nutritional assessment, counselling and support to provide a balanced diet with adequate energy, and food supplementation for severely malnourished individuals, or for individuals in food-insecure areas.^1^, ^9^ Thus, typically, national guidelines currently recommend screening individuals with their BMI and providing nutritional supplementation selectively to individuals defined as severely malnourished. In sub-Saharan Africa, national guidelines vary widely in their recommendations on provision of ready-to-use lipid-based nutritional supplements;^10^ however, they are increasingly being given within HIV programmes in the region.^11^

Lipid-based supplementary foods have been highlighted as a key potential intervention to reduce mortality in severely immunocompromised HIV-infected individuals by improving both nutritional status and antiretroviral drug absorption.^12^ There are several mechanisms by which such supplementary foods could have beneficial effects on mortality. For example, improved nutrition could have direct effects on mucosal barriers and immune function, reducing inflammation and thereby reducing the nuclear factor κ-light-chain-enhancer of activated B cell-induced enhancement of viral replication. Alternatively, improved strength and wellbeing and less hunger could improve ART adherence, or enhanced nutritional intake could improve drug absorption and metabolism directly. However, lipid-based supplementary foods are more expensive than other forms of macronutrient supplementation or nutritional counselling alone. Meta-analyses of trials of food supplementation or macronutrient supplements across a range of HIV-disease stages and stages of undernutrition have reported inconclusive evidence on mortality, virological, immunological, and functional outcomes.^13^, ^14^

We therefore did the Reduction of EArly mortaLITY (REALITY) trial in four sub-Saharan African countries to investigate whether universally providing ready-to-use supplementary food (RUSF) to improve nutritional status after ART initiation reduced early mortality in severely immunocompromised HIV-infected adults and children aged at least 5 years. We report the effect of universal provision of nutritional supplementation on mortality, disease progression, and anthropometry among these severely immunocompromised individuals at ART initiation compared with the current standard of care of providing nutritional intervention only to individuals identified as severely malnourished.

## Methods

### Study design and participants

We did a 2 × 2 × 2 factorial, open-label, parallel-group trial at inpatient and outpatient facilities in eight urban or periurban regional hospitals in Kenya, Malawi, Uganda, and Zimbabwe. The trial was approved by ethics committees in Kenya (Moi University Institutional Research and Ethics Committee and the Kenya Medical Research Institute Ethics Review Committee), Malawi (College of Medicine Research Ethics Committee), Uganda (Joint Clinical Research Centre Institutional Review Board and the Uganda National Council for Science and Technology), the UK (University College London Ethics Committee), the USA (Indiana University Institutional Review Board), and Zimbabwe (Joint Parirenyatwa Hospital and College of Health Sciences Research Ethics Committee and the Medical Research Council of Zimbabwe).

We consecutively approached adults and children aged at least 5 years with a diagnosis of HIV infection identified through national screening programmes, who were not on ART and reported no previous ART, and were initiating ART at one of the facilities, with CD4 counts of fewer than 100 cells per μL, for screening. In centres where CD4 cell counts were not routinely done at diagnosis, patients with a new diagnosis of HIV were approached consecutively for CD4 testing and study screening. Exclusion criteria were pregnancy or breastfeeding, use of single-dose nevirapine to prevent mother-to-child-transmission of HIV, and any contraindications to nutritional supplementation or trial drugs. Adults and children's guardians gave written informed consent; older children gave additional assent following national guidelines.

### Randomisation and masking

We randomly assigned participants (1:1) to initiate ART with or without universal provision of 12 weeks of peanut-based RUSF providing 1000 kcal/day and micronutrients as two 92 g foil packets daily (children aged 5–12 years received one 92 g foil packet of RUSF daily, providing 500 kcal per day; appendix p 2). This was designed to replicate the way that supplementary foods are typically used in practice and in line with recent trials,^15^, ^16^, ^17^ rather than individually targeting estimates of individuals' energy gap. In both groups, those identified as malnourished according to national criteria (generally BMI <16–18 kg/m^2^, or for children, measures equivalent to BMI-for-age *Z* scores <–3; appendix pp 2–3) were referred to the local nutrition programme for ready-to-use therapeutic food (RUTF) whenever it was available, as normally done in routine care. RUTF has similar composition to RUSF, but might be prescribed in different quantities. If RUTF was not available, malnourished participants randomly assigned to RUSF received that instead; those assigned to no-RUSF did not receive any supplementation in accordance with local clinical practice. Therefore, the randomisation compared universal provision of nutritional supplementation with RUSF or RUTF (RUSF) with the selective provision of RUTF only for malnourished individuals (no-RUSF).

We also tested two other separate interventions using a factorial 2 × 2 × 2 design; participants were also randomly assigned (1:1) to 12 weeks of ART intensification with raltegravir versus no added raltegravir, and to enhanced infective prophylaxis^18^ versus standard co-trimoxazole prophylaxis (appendix pp 2–3). These other randomised interventions are reported separately. Randomisation was stratified by centre, age (up to 12 years or 13 years and older), and the other factorial randomisations. A computer-generated sequential randomisation list with permuted blocks of different sizes was prepared by the trial statistician and incorporated securely into the online trial database. The list was concealed until allocation, after eligibility was confirmed by local centre staff who then did the randomisation. Nurses and physicians assessing outcomes were not masked to group assignment, but information on the randomised allocation was not available to staff assaying laboratory tests.

### Procedures

All participants initiated ART with two nucleoside-reverse-transcriptase-inhibitors and one non-nucleoside-reverse-transcriptase-inhibitor. RUSF was purchased from Valid International. Clinic visits were scheduled at weeks 2, 4, 8, 12, 18, 24, 36, and 48, and included nurse assessment of vital status and symptoms by use of a standardised checklist, self-reported adherence assessment (including any missed doses in the previous 4 weeks) by participants or children's carers, and dispensing of all medication including ART and RUSF. Standard adherence support was provided following local clinic practice. Food insecurity was assessed by self-report at baseline (by asking whether there was enough food to provide everyone in the household with regular meals). Body composition and basal metabolic rate were assessed at these visits by bioimpedance analysis with a Tanita BC-420MA machine. At enrolment and weeks 4, 12, and 48, grip strength was measured with a Takei 5401 Hand Grip Dynamometer. In Zimbabwe only, two body circumferences and four skinfold thicknesses were measured at enrolment and weeks 12 and 48 (appendix p 4). At enrolment and weeks 4, 12, 24, 36, and 48, history was taken and examination was done by physicians; haematology, biochemistry (weeks 0, 4 and 48 only), and CD4 cell counts were done; and plasma was stored for retrospective measurement of HIV viral load (results were not available in real time). Toxicity substitution or second-line treatment switches were at physicians' discretion, following WHO guidelines.^19^ Participants exited the trial after 48 weeks.

### Outcomes

The primary outcome was all-cause mortality to 24 weeks. Secondary outcomes assessed at 48 weeks were all-cause mortality; serious adverse events, grade 4 adverse events, and adverse events leading to modification of ART or trial drugs;^20^, ^21^ absolute changes in weight, mean upper-arm circumference (MUAC), and BMI; viral load; CD4 cell counts; incidence of new diagnoses of tuberculosis, cryptococcal disease, candidiasis, or severe bacterial infections; admissions to hospital; self-reported adherence (reporting any missed doses in the previous 4 weeks) to RUSF and ART, and acceptability of RUSF. Secondary clinical endpoints, cause of death and trial-drug relatedness were adjudicated against protocol-defined criteria by an endpoint review committee (most of whom were independent members), masked to trial interventions received, using available clinical or laboratory data; this committee also adjudicated whether events were newly occurring during the trial or had been pre-existing at baseline. We assessed mortality at 12 weeks in an exploratory analysis. Loss to follow-up was defined as no clinic attendance for more than 90 days.

### Statistical analysis

We calculated that 1800 adults and children would provide more than 80% power to detect a 50% relative reduction in 24 week all-cause mortality from 7% to 3·5% (two-sided α=0·05), allowing for a 5% loss to follow-up and a single inflation factor to allow for the factorial design. The effect size was based on achieving a clinically meaningful absolute reduction in mortality for at least one of the three randomisations.

Interim data were reviewed by an independent data monitoring committee (in three annual meetings) with the Haybittle-Peto criterion (p<0·001). Randomised groups were compared following intention to treat with log-rank tests for time-to-event outcomes (including mortality and time-to-new-disease events), Fisher's exact tests for binary outcomes, Poisson models for counts of admissions to hospital, and generalised estimating equations with independent working correlation for global tests of repeated measures (normal distribution for BMI, weight, CD4 cell counts, and other continuous outcomes; and logistic distribution for self-reported adherence and suppression of viral load). Primary analyses were stratified by the randomisation stratification factors (including stratification factors and scheduled visit week as categorical independent variables in the generalised estimating equations). Prespecified subgroup analyses were done by the other factorial randomisations, age, site, country, baseline CD4 cell count, type of ART, and BMI (appendix pp 4–5). Where data were missing, denominators are given and analysis was based on complete- lated based on days prescribed, but excluding the time after participants or physicians reported that it had been permanently discontinued. This outcome also excluded any time participants reported they had not taken it completely, and occurring any time after prescriptions would have finished (eg, due to a late return to clinic). This calculation did not incorporate any adjustment for self-reported non-adherence. We analysed the data with Stata version 15.1. This trial is registered with ClinicalTrials.gov (NCT01825031) and the ISRCTN registry (43622374).

### Role of the funding source

The funder of the study had no role in study design, data collection, data analysis, data interpretation, or writing of the report. The corresponding authors had full access to all the data in the study and had final responsibility for the decision to submit for publication.

## Results

**Figure 1:**
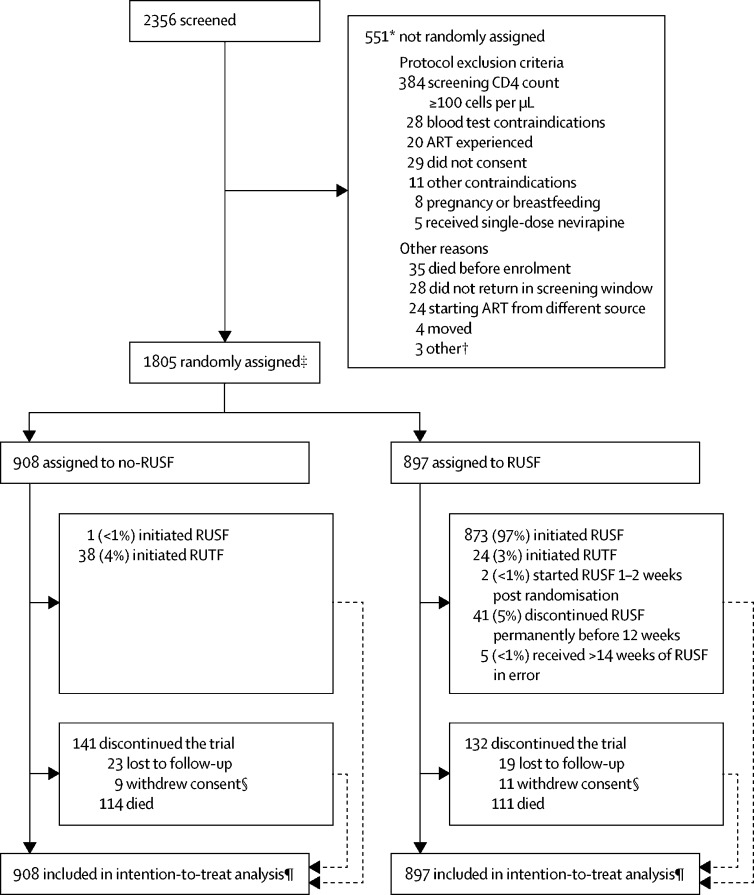
Trial profile ART=antiretroviral therapy. RUSF=ready-to-use supplementary food. RUTF=ready-to-use therapeutic food. *Reasons were not mutually exclusive, therefore total is more than the number of patients not randomly assigned treatment. †Considered too unwell (one patient), not able to comply with trial schedule (one patient), and no further details (one patient). ‡Ten patients ineligible after randomisation (four previously received ART, one RUSF contraindicated [milk allergy]), one 3 months pregnant, one had CD4 count ≥100 cells per μL at screening [38 cells per μL at enrolment], one randomly assigned 7 weeks after screening, two incorrect consent [one aged 14 years gave assent but without caregiver consent at enrolment; one aged 19 years old gave assent but caregiver consent was obtained on the basis of a self-reported age of 16 years at screening]). §Four patients assigned no-RUSF and two assigned RUSF were not formally lost to follow-up (they were seen in the clinic within 91 days of week 48). ¶Time-to-event analyses included all times at-risk from randomisation to the earliest of the event or last clinical follow-up if the event had not occurred (details on adherence to randomised strategy in appendix).

Between June 18, 2013, and April 10, 2015, we randomly assigned 1805 participants to treatment, 897 to RUSF and 908 to no-RUSF (figure 1), who were included in the analyses. 28 participants (3%) from each group were lost to follow-up (p=1·00). 104 (12%) assigned RUSF versus 108 (12%) no-RUSF missed at least one scheduled visit before death or loss to follow-up (p=0·84). Baseline characteristics were well balanced between groups (table, appendix p 6).

**Table:** Baseline characteristics of the intention-to-treat population

		**No-RUSF (n=908)**	**RUSF (n=897)**	**All (n=1805)**
Male sex		487 (54%)	474 (53%)	961 (53%)
Age at last birthday (years)		36 (29–42; 5–71)	36 (29–42; 6–77)	36 (29–42; 5–77)
	5–12	23 (3%)	17 (2%)	40 (2%)
	13–17	18 (2%)	14 (2%)	32 (2%)
WHO HIV stage				
	1	152 (17%)	148 (16%)	300 (17%)
	2	293 (32%)	261 (29%)	554 (31%)
	3	338 (37%)	353 (39%)	691 (38%)
	4	125 (14%)	135 (15%)	260 (14%)
In individuals aged ≥13 years				
	Weight* (kg)† (n=1760)	52·7 (46·0–59·5)	52·4 (46·5–59·2)	52·5 (46·3–59·3)
	BMI* (kg/m^2^)† (n=1758)	19·3 (17·3–21·6)	19·2 (17·4–21·4)	19·3 (17·4–21·5)
	BMI <18·5 kg/m^2^	354/880 (40·2%)	343/878 (39%)	697/1758 (40%)
	MUAC* (cm; n=1764)	24·0 (21·8–26·2)	24·0 (22·0–26·0)	24·0 (22·0–26·1)
	Lean body mass (kg; n=1704)	41·4 (37·4–46·9)	41·5 (37·2–47·1)	41·5 (37·3–47·0)
	Fat mass (kg; n=1707)	6·4 (3·5–12·3)	6·8 (3·8–12·3)	6·6 (3·7–12·3)
	Basal metabolic rate (kcal/day; n=1674)	1305 (1193–1438)	1300 (1192–1446)	1303 (1192–1441)
Grip strength (k; n=1772)		24·2 (19·2–30·9)	24·7 (19·4–31·1)	24·5 (19·3–31·0)
Household food reported sufficient to feed		627/898 (70%)	610/890 (69%)	1237/1788 (69%)
Household reported to grow own crops		489/897 (55%)	475/888 (53%)	964/1785 (54%)
CD4 count* (cells per μL)		34 (16–60)	38 (16–64)	37 (16–63)
	0–24	344 (38%)	312 (35%)	656 (36%)
	25–49	258 (28%)	251 (28%)	509 (28%)
HIV viral load (copies per mL; n=1804)		244 110 (94 550–589 300)	251 680 (95 680–626 610)	249 770 (95 280–606 360)
	≥100 000	675/908 (74%)	659/896 (74%)	1334/1804 (74%)
	<1000‡	8/908 (<1%)	6/896 (<1%)	14/1804 (<1%)
Haemoglobin (g/L; n=1800)		111 (96–126)	112 (95–128)	112 (96–127)
Supplementation prescribed at randomisation				
	RUSF§	1 (<1%)	873 (97%)	874 (48%)
	RUTF	38 (4%)	24 (3%)	62 (3%)
	No supplementation	869 (96%)	0	869 (48%)

Data are n (%), median (IQR; range), or median (IQR). RUSF=ready-to-use supplementary food. BMI=body-mass index. MUAC=mid-upper-arm-circumference. RUTF=ready-to-use therapeutic food.

* Mean of screening and enrolment values. For eligibility, screening CD4 count had to be <100 cells per μL, so baseline values might be more than 100 cells per μL depending on the count at enrolment.

† Among children aged younger than 13 years, median (IQR) weight-for-age, height-for-age, and BMI-for-age *Z* scores were −2·6 (−3·4 to −1·5), −1·9 (−2·6 to −1·3), and −1·9 (−2·8 to −1·1), respectively.

‡ Potentially indicating undisclosed previous antiretroviral therapy: median CD4 cell count of 76 cells per μL in these participants.

§ One participant with BMI 17·8 kg/m^2^ randomly assigned to no-RUSF started RUSF on day 0 (protocol deviation).

All participants assigned RUSF started either RUSF or RUTF; one (<1%) individual assigned no-RUSF started RUSF in error (table, figure 1). Of 111 no-RUSF participants with BMI less than 16 kg/m^2^, 27 (24%) were prescribed RUTF (appendix p 7). In the first 12 weeks on ART, the RUSF group spent 95% person-time prescribed RUSF and 2% person-time prescribed RUTF versus 2% and 3% respectively in the no-RUSF group (appendix p 19). After 12 weeks, 2% and <1% person-time was spent prescribed RUSF and RUTF, respectively, in the RUSF group, and 1% and <1% person-time, respectively, in the no-RUSF group. 207 participants (27%) of 769 in the RUSF group reported missing any RUSF at weeks 0–4 and 129 (19%) of 685 at weeks 8–12 (appendix p 20). 110 participants (14%) of 795 in the RUSF group reported difficulty taking RUSF every day as prescribed at 4 weeks, and 59 (8%) of 734 reported difficulty at 12 weeks (appendix p 20).

**Figure 2:**
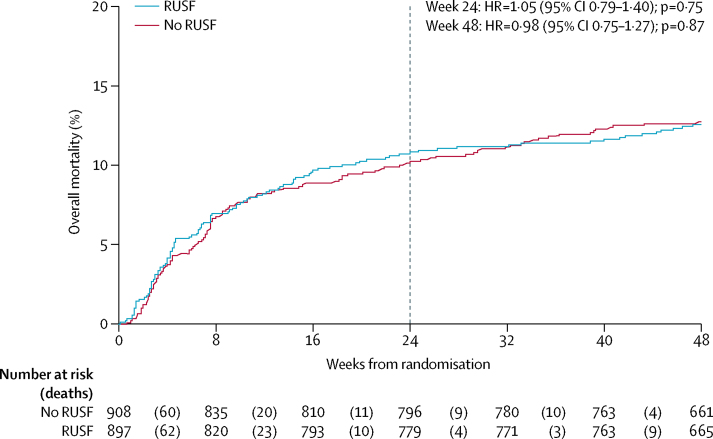
Overall mortality through 48 weeks Dotted vertical line at week 24 when the primary outcome (mortality) was measured. HR=hazard ratio. RUSF=ready-to-use supplementary food.

By 24 weeks, 96 participants (Kaplan-Meier percentage 10·9%, 95% CI 9·0–13·1) allocated to RUSF and 92 (10·3%, 8·5–12·5) to no-RUSF died (figure 2, hazard ratio [HR] 1·05, 95% CI 0·79–1·40; log-rank p=0·75). By 48 weeks 111 (12·6%, 10·6–15·0) and 114 (12·8%, 10·8–15·2) died (0·98, 0·75–1·27; p=0·87). By 12 weeks (exploratory analysis), 73 (8·2%) and 74 (8·2%) died (0·99, 0·72–1·37; p=0·96). There was no evidence of interaction with other factorial randomisations (p>0·7). Causes of death were similar between the two groups (p_exact_=0·75; appendix p 7). There was some evidence that RUSF could be harmful in participants in one of the two Kenyan centres (p_interaction_=0·02), but no evidence (p>0·1) that mortality differences varied across eight other preplanned or seven other exploratory subgroups (total 15 analyses; one p value <0·05 expected by chance; appendix p 22). In particular, there was no evidence that the effect of RUSF varied by BMI at ART initiation (p=0·47, 0·45, and 0·50 using continuous, categorical [of threshold 20 kg/m^2^], or categorical [of threshold 18·5kg/m^2^] interactions) or by reported household food insecurity (p=0·51) or food crop growth (p=0·20; appendix p 22).

There was no evidence of an effect of RUSF on disease progression (new WHO stage 4 or death p=0·99, new WHO stage 3 or 4 or death p=0·86), new tuberculosis disease (p=0·91), cryptococcal disease (p=0·96), candidiasis (p=0·83), or presumptive severe bacterial infections (p=0·45; appendix p 8). There was also no evidence for differences in time to first serious adverse events, which occurred in 205 participants (23%) assigned RUSF and 205 (23%) assigned no-RUSF (p=0·81; appendix pp 8–17). The most common type of serious adverse event was specific infections, occurring in 90 (10%) of 897 participants assigned RUSF and 87 (10%) of 908 assigned no-RUSF. There was also no evidence of differences in grade 4 adverse events that occurred in 181 (20%) and 172 (19%; p=0·45), new admissions to hospital that occurred in 169 (19%) and 171 (19%; p=0·99), or adverse events of grade 3 or 4 that occurred in 327 (36%) and 331 (36%; p=0·90; appendix p 8). Total number of days admitted to hospital were 2342 with no-RUSF versus 2710 with RUSF (rank-sum p=0·66), from 209 versus 222 admissions, respectively (Poisson p=0·44). Adverse events were reported as the reason for stopping RUSF (permanently or temporarily, regardless of duration) in 46 participants (5%; appendix p 18), mainly gastrointestinal events.

Self-reported ART adherence did not differ between randomised groups (global p=0·81, appendix p 21). Consistent with this finding, there were no differences at any timepoint between randomised groups in suppression of viral load to fewer than 50 copies per mL (global p=0·58, appendix p 23) or change in CD4 cell count (p=0·34, appendix p 23). 48 weeks after ART initiation, 603 participants (81%) of 749 assigned RUSF versus 599 (79%) of 754 assigned to no-RUSF had a viral load of fewer than 50 copies per mL (p=0·61), and the mean increases in CD4 count were 154 cells per μL (SD 4·3) and 155 cells per μL (4·5), respectively (p=0·87; appendix p 23).

**Figure 3:**
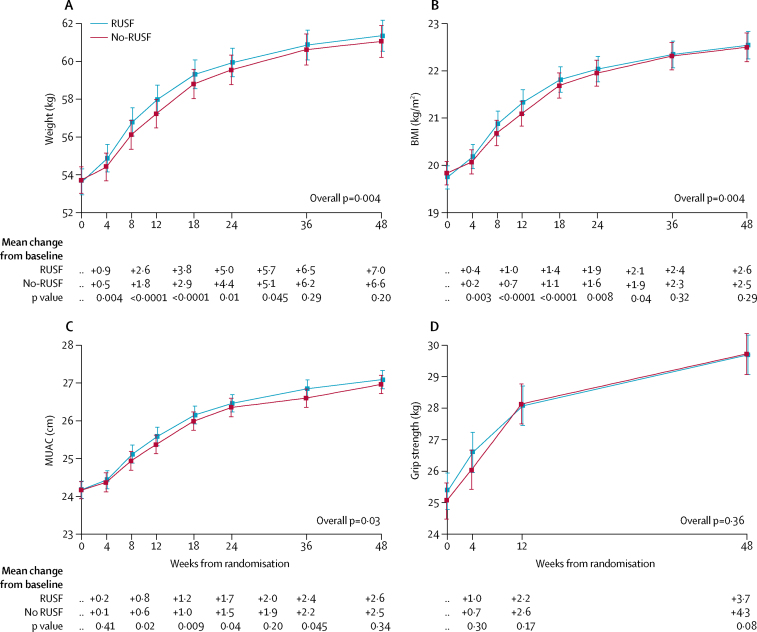
Changes in weight, BMI, MUAC, and grip strength hrough 48 weeks Data are mean (95% CI). Figure shows changes in (A) weight, (B) body-mass index (BMI), (C) MUAC (mid-upper-arm-circumference), and (D) grip strength. p values compare changes from baseline across randomised groups, and hence adjust for any imbalances at baseline. Weight, BMI, and MUAC were analysed only in those aged ≥13 years at initiation of antiretroviral therapy. RUSF=ready-to-use supplementary food.

**Figure 4:**
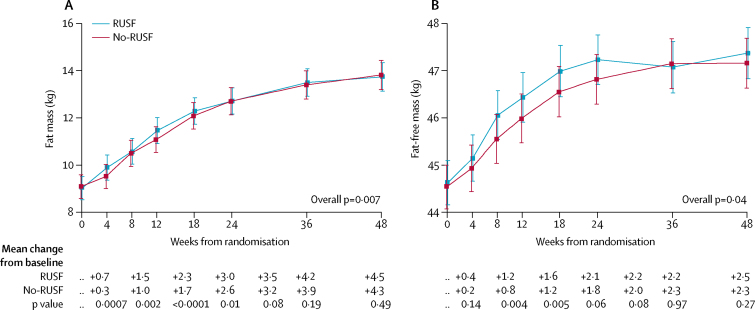
Changes in body composition through 48 weeks Data are mean (95% CI) for patients aged ≥13 years at initiation of antiretroviral therapy. Figure shows changes in (A) fat mass and (B) fat-free mass. p values compare changes from baseline across randomly assigned groups and hence adjust for any imbalances at baseline. RUSF=ready-to-use supplementary food.

Participants aged at least 13 years who were assigned RUSF had significantly greater gains in weight, BMI, and MUAC through 48 weeks than those assigned no-RUSF (overall p=0·004, 0·004, and 0·03, respectively; figure 3). Differences between randomised groups were greatest at 12 weeks (figure 3). There was no evidence that the greater anthropometric increases in the RUSF group varied according to BMI at ART initiation (appendix p 24). In adults and adolescents, estimates from bioimpedance analysis suggested that at 12 weeks, in those receiving RUSF, the additional weight gain over the no-RUSF group comprised of both fat mass (p<0·0001) and fat-free mass (p=0·005; figure 4).

Among Zimbabwean adults and adolescent participants, waist-circumference, hip-circumference, suprailiac skinfold thickness, and the sum-of-four skinfold thicknesses (triceps, subscapular, suprailiac, and mid-thigh) increased significantly more in those assigned RUSF than in those assigned no-RUSF at 12 weeks (p<0·04, appendix pp 25–28), but differences in triceps, subscapular, and mid-thigh skinfold thicknesses were not significant (p=0·14–0·21). We observed no evidence of sustained differences between randomised groups through 48 weeks (global p>0·10). In 40 participants younger than 13 years, there were no differences in the changes in weight-for-age or BMI-for-age *Z* scores between groups (appendix p 29).

In all participants, grip strength increased after starting ART but changes in grip strength did not differ between groups (p=0·36; figure 3), with increases at 12 weeks of 2·2 kg (SD 0·2) in RUSF versus 2·6 kg (0·2) in no-RUSF (p=0·17). There were also no significant differences between randomised groups for changes in haemoglobin (p=0·88).

## Discussion

Among severely immunocompromised individuals initiating ART in Africa, universal provision of RUSF did not reduce early mortality or change suppression of viral load, CD4 cell count recovery, ART adherence, serious morbidity events, or recovery of grip strength compared with following current guidelines for selective nutritional supplementation in severely malnourished patients (received by around 4% of the no-RUSF group). Universal RUSF was associated with increased gains in weight, BMI, and MUAC during the supplementation period that were not sustained afterwards. The large size of the REALITY trial, and similar mortality risks between the RUSF and non-RUSF groups, does not suggest that the null result was because of an absence of statistical power. In fact, 24-week mortality was higher than anticipated (10·5% rather than 7%), meaning that the trial had 80% power to detect reductions from 10·5% to 6·5% (HR 0·6). There was no evidence of effect modification by baseline BMI or self-reported household food insecurity.

Few randomised trials of nutritional supplementation in HIV-infected individuals have been designed to assess a primary endpoint of mortality or have included an unsupplemented control group, aside from treatment of severe malnutrition. Instead, nutritional supplementation is commonly assumed to be beneficial, probably because in observational studies, weight gain among malnourished individuals after starting ART has been associated with better outcomes.^22^ However, besides the substantial cost implications of lipid-based nutritional supplements, individuals starting ART are at risk of metabolic abnormalities including insulin resistance and dyslipidaemia,^23^ and there is increasing concern over the long-term effects in other contexts where rapid weight gain is achieved through lipid-based nutritional supplements.

Two trials of nutritional supplementation among malnourished individuals starting ART have reported mortality endpoints.^15^, ^16^ The NUSTART trial^15^ in Tanzania and Zambia enrolled 1876 adults with BMI less than 18·5 kg/m^2^ eligible for ART with CD4 counts fewer than 350 cells per μL or stage 3 or 4 HIV disease. Participants were randomised to receive a lipid-based nutritional supplement with or without additional vitamins and minerals (150 kcal per day for 2 weeks, then 1400 kcal per day) from referral for ART until 6 weeks after starting ART. Participants were more malnourished than in our trial (mean BMI 16·4 kg/m^2^), although their mean CD4 cell count was higher (around 135 cells per μL). 365 participants (19%) died 12 weeks after starting ART, with no differences in mortality between randomised groups (rate ratio 0·99, 95% CI 0·80–1·21), and no differences in changes in BMI or incidence of serious adverse events. Those allocated RUSF with micronutrients had a modestly greater increase in CD4 cell count. In the second trial,^16^ 491 Malawian adult men with BMI less than 18·5 kg/m^2^ were randomly assigned at ART initiation to receive either RUSF or a blend of corn and soy, both providing 1360 kcal per day. Mean baseline BMI and CD4 counts were similar to those in the NUSTART trial: 16·5 kg/m^2^ and 131–142 cells per μL). Participants allocated RUSF gained more BMI and lean mass than those allocated the blend of corn and soy, but there was no difference between groups at study end in participants categorised as moderately or severely malnourished. Mortality to 14 weeks was similar in the two groups (26% and 27%). At 12 months, both groups had similar BMI, fat-free body mass, number of admissions to hospital, and mortality.

Another trial (ARTFood)^17^ in Ethiopia included an unsupplemented control group, but was not designed to assess mortality. In this study, 282 adults initiating ART with BMI greater than 17 kg/m^2^ were randomly assigned either early supplementation with immediate whey-based or soy-based RUSF (1100 kcal per day for 3 months), or delayed supplementation, 3 months after ART initiation. The median baseline BMI (19·8–20·0 kg/m^2^) was similar to that in our trial, but the median CD4 counts were higher (181–191 cells per μL). Early RUSF supplementation was associated with gains in weight and lean mass (+2·1 kg total, +0·9 kg lean body-mass; BMI changes not reported) and a small increase in grip strength. There were no differences in viral load or CD4 cell counts and only four participants were reported to have died. At 12 months, there was no difference in weight gain between groups.

Compared with the ARTFood trial, in REALITY we enrolled only profoundly immunocompromised individuals at much higher risk of mortality. The differential short-term weight gain with RUSF (+0·9 kg total; +0·4 kg fat-free mass) was somewhat smaller than that noted in the ARTFood trial. We also showed, unlike in ARTFood, that grip strength improved irrespective of allocation to RUSF. These two findings probably reflect differences between the participants in the ARTFood and REALITY trials. Any wasting and weakness in our trial might have been more because of the effects of HIV (as indicated by their severely immunocompromised status) than primary undernutrition,^22^ with corresponding differences in the metabolic responses to ART and nutritional supplementation. Additionally, although only 40% of participants in our trial had BMI less than 18·5 kg/m^2^, we noted no evidence that the effect of RUSF varied by BMI at baseline, whether this was considered as a continuous factor, or when stratified by common thresholds to define malnutrition or undernutrition (ie, 18·5 or 20·0 kg/m^2^).

In 2015, a systematic review^14^ of nutritional assessment, counselling, and support in people living with HIV did not identify any further trials with a mortality endpoint. The review concluded that studies were of variable and largely poor quality and the effect of nutritional supplementation on mortality, morbidity, and disease outcomes was uncertain.^14^ In 2013, a Cochrane review^13^ identified 11 trials including a total of 1725 HIV-infected adults with different disease stages, ART status, and nutritional status, comparing various supplements with placebo, no supplementation, nutrition counselling alone, or usual diet. No trials were identified assessing the effect of macronutrient interventions on mortality as a primary outcome. Meta-analysis showed a significant effect of supplementation on increasing energy and protein intake, but no evidence of efficacy of nutritional supplementation on clinical, anthropometric, or immunological outcomes.^13^

The major strengths of our trial were its large size, the mortality primary endpoint in a well defined, high-risk population, and having a non-supplemented comparison group aside from a small proportion who were severely malnourished. For generalisability, we included a large number of participants at eight HIV clinics across four African countries. Our limitations included that the results cannot inform on the potential effect of RUSF provision on retention in clinic follow-up in a real-world setting since the design included defaulter tracing because this was a clinical trial. Other limitations included the absence of precise data on actual home use or sharing of RUSF, and on feeding frequency and dietary diversity. We also recruited fewer children than planned, limiting our ability to assess effects in this subgroup; we planned to enrol 300 children older than 5 years but found few children with CD4 cell counts less than 100 cells per μL, potentially as a result of increasing coverage of ART to prevent mother-to-child HIV transmission. By contrast, we recruited adults much faster than expected, suggesting trial results are generalisable to the population presenting with low CD4 cell counts.

Consistent with results from previous trials, those from our REALITY trial showed that increasing weight gain by universal lipid-based supplementation, which occurred across all baseline BMI subgroups, did not lead to reductions in mortality. This finding suggests that there is not a direct causal relation between BMI and mortality. Instead, the main drivers of mortality seem to be opportunistic infections as a result of immuno-suppression and systemic immune activation,^24^ which, together with reduced appetite and feeding or swallowing difficulties, lead to low BMI, and might not be directly improved by nutritional rehabilitation.^25^ Besides reducing viral load and preventing opportunistic infections, new strategies to reduce immune activation might improve outcomes.^25^, ^26^ Systemic inflammation in HIV particularly arises from the gastrointestinal tract,^26^, ^27^ thus interventions targeting gut dysfunction, inflammation, microbial translocation, and the microbiota are promising targets for further research.^28^

Both undernutrition and nutritional supplementation have been reported to change the pharmacokinetics of ART. In one study of Ugandan women,^29^ exposure to lopinavir, ritonavir, and efavirenz was lower in those who were undernourished, ascribed to decreased bioavailability. Fat might increase efavirenz absorption; however, in another study of Ethiopian adults,^30^ allocation to lipid-based nutritional supplementation did not affect efavirenz plasma concentrations, although plasma nevirapine concentrations were lower. In our trial, ART regimens were well balanced across the groups (appendix p 6) and we showed no evidence of differences in viral load suppression, thus there was no suggestion of clinically significant changes in the bioavailability of antiretroviral drugs with RUSF.

Among severely immunocompromised adults and older children initiating ART, the REALITY trial provides evidence that universally providing lipid-based RUSF increases bodyweight in the short-term, but does not improve mortality, disease progression, viral load, or immunological recovery compared with selectively prescribing RUSF or RUTF to severely malnourished individuals according to current protocols. Although individuals with severe malnutrition should continue to be identified and treated, a change in policy to provide lipid-based nutritional supplements to all severely immunocompromised individuals starting ART is not warranted.

**This online publication has been corrected. The corrected version first appeared at thelancet.com/hiv on May 29, 2018**
