# Corrections

**DOI:** 10.1016/S2352-3018(18)30113-9

**Published:** 2018-05-29

*Mallewa J, Szubert AJ, Mugyenyi P, et al. Effect of ready-to-use supplementary food on mortality in severely immunocompromised HIV-infected individuals in Africa initiating antiretroviral therapy (REALITY): an open-label, parallel-group, randomised controlled trial.* Lancet HIV *2018; **5**: e231–40*—The open access license for this paper has been amended to CC BY 4.0. This correction has been made to the online version as of May 29, 2018.

