# Supplementary material for: Effect of ready-to-use supplementary food on mortality in severely immunocompromised HIV-infected individuals in Africa initiating antiretroviral therapy (REALITY): an open-label, parallel-group, randomised controlled trial
Source: Lancet HIV. 2018 Apr 10;5(5):e231–40. doi: 10.1016/S2352-3018(18)30038-9 (PMC5932190; doi:10.1016/S2352-3018(18)30038-9)

THE LANCET
HIV

Supplementary appendix

This appendix formed part of the original submission and has been peer reviewed. We post it as supplied by the authors.

Supplement to: Mallewa J, Szubert AJ, Mugyenyi P, et al, on behalf of the REALITY trial team. Effect of ready-to-use supplementary food on mortality in severely immunocompromised HIV-infected individuals in Africa initiating antiretroviral therapy (REALITY): an open-label, parallel-group, randomised controlled trial. *Lancet HIV* 2018; published online April 10. http://dx.doi.org/10.1016/S2352-3018(18)30038-9.

Online Only Supplement to:

Ready to Use Supplementary Food to Prevent Mortality in Severely Immunocompromized Individuals Initiating Antiretroviral Therapy in Africa: An Open-label Randomised Controlled Trial

Methods **2**

(a) Study sites 2

(b) RUSF composition 2

(c) Further details of RUSF randomisation 2

(d) Antiretroviral therapy and raltegravir randomisation 3

(e) Further details of enhanced prophylaxis randomisation 3

(f) Further details of endpoint ascertainment 3

(g) qBody circumference and skinfold thicknesses 4

(h) Sample size calculation from the trial protocol 4

(i) Statistical methods 4

(j) Subgroup analyses 4

References for supplementary material **5**

Supplementary Tables **6**

(k) Supplementary Table 1: Additional baseline characteristics and ART received 6

(l) Supplementary Table 2: Receipt of RUSF and RUTF according to baseline BMI 7

(m) Supplementary Table 3: Causes of death through 48 weeks 7

(n) Supplementary Table 4: Secondary and other outcomes through 48 weeks 8

(o) Supplementary Table 5: Serious adverse events 9

(p) Supplementary Table 6: Adverse events and poor acceptability leading to discontinuation of RUSF ...18

Supplementary Figures **19**

(q) Supplementary Figure 1: RUSF and RUTF received over time 19

(r) Supplementary Figure 2: Self-reported adherence to RUSF and ART, and acceptability 20

(s) Supplementary Figure 3: Subgroup analyses for primary endpoint (24-week mortality) 22

(t) Supplementary Figure 4: HIV viral load, CD4 cell count and ART adherence 23

(u) Supplementary Figure 5: (a) Mean BMI and (b) changes in BMI (kg/m^2) according to BMI at ART initiation 24

(v) Supplementary Figure 6: Changes in body-circumferences and skinfold thicknesses (participants from Harare only) 25

(w) Supplementary Figure 7: Weight and BMI-for-age Z score changes for participants aged <13 years (N=40; 23 no-RUSF, 17 RUSF) 29

Methods

(a) Study sites

The REALITY (Reducing EArly mortaLITY) trial recruited adults and older children from Zimbabwe (University of Zimbabwe Clinical Research Centre), Uganda (Joint Clinical Research Centre sites in Mbarara, Mbale, Gulu and Fort Portal, overseen by Kampala), Malawi (Department/College of Medicine and Malawi-Liverpool-Wellcome Trust Clinical Research Programme, Blantyre), and Kenya (KEMRI Wellcome Trust Research Programme and Academic Model for the Prevention and Treatment of HIV/AIDS (AMPATH) Centre at Moi Teaching Referral Hospital (MTRH)). These centres had previously participated in trials in HIV-infected adults and children and so could conduct the trial to a high standard.

(b) RUSF composition

Ingredients: Peanut Paste, Skimmed Milk Powder, Vegetable Oils & Fat, Sugar, Vitamins & Minerals, Stabiliser: Vegetable Monoglycerides.
Macronutrients: 500kcal per 92g sachet, Protein 10-12% and Lipids 45-60% of total calories.
Micronutrients:

	Unit	Per 92g Sachet	Per two sachets daily ration
Biotin	µg	55	110
Niacin	mg	4.6	9.2
Folic acid	µg	184	368
Pantothenic acid	mg	2.8	5.6
Vitamin A	µg<br>IU	875<br>2910	1750<br>5820
Vitamin B1	mg	0.5	1.0
Vitamin B12	µg	1.5	3.0
Vitamin B2	mg	1.5	3.0
Vitamin B6	mg	0.6	1.2
Vitamin C	mg	46	92
Vitamin D	µg	16.1	32.2
Vitamin E	mg	18.4	36.6
Vitamin K	µg	20.7	41.4
Calcium	mg	415	830
Copper	mg	1.5	3.0
Iodine	µg	97	194
Iron	mg	11	22
Magnesium	mg	100	200
Phosphorus (excluding phytate)	mg	415	830
Potassium	mg	1150	2300
Selenium	µg	28	56
Sodium (maximum)	mg	265	530
Zinc	mg	12	23

(c) Further details of RUSF randomisation

Standard-of-care nutritional support followed current local practice in each centre, based on criteria for body mass index (BMI) and/or mid-upper arm circumference (MUAC), and the availability of ready-to-use therapeutic food (RUTF) supplements through the national programmes at the time of randomization (see table below for recommendations in place at the start of the trial). Standard management of malnutrition initiating ART varied across centres since national guidelines differed between countries: most were provided with micronutrients and advice on diet, whilst RUTF was provided to severely malnourished adults with BMI<16-18 kg/m^2 or MUAC<16cm at some centres, when it was available locally. Adults and children with mild-moderate malnutrition did not receive any nutritional support following national guidelines at the time the trial was recruiting, except in Kilifi where those with BMI<18.5 kg/m^2 received Plumpy' Soy or Acha Mum (2 sachets daily). Participants eligible for or receiving food products from national programs transitioned onto the study product when they finished it if randomised to the enhanced nutritional support and still <12 weeks from randomisation.

Country	Adults/adolescents: eligibility for therapeutic feeding	Children: eligibility for therapeutic feeding in children aged 5 years or older
Kenya	Kilifi: BMI<16<br>Eldoret: BMI<16 or MUAC<16cm	Weight-for-height z-score<-3
Malawi	BMI<17	MUAC<12cm, weight<80% expected for height
Uganda	BMI<18	Weight for height < 70% for children of all ages<br>MUAC <11.5 cm for children 6months to <6yrs<br>MUAC <13.5 cm for children 6yrs to <10yrs<br>MUAC <15.7 cm for children 10yrs and older
Zimbabwe	BMI<17	BMI for age z-score<-3

Note: as per the approved protocol. Some guidelines changed during the course of the trial, and not all eligible patients received RUTF if it was not available through national programmes at the time of randomisation.

(d) Antiretroviral therapy and raltegravir randomisation

Participants initiated ART a median 5 days after screening. Adults predominantly started first-line tenofovir+emtricitabine+efavirenz, or zidovudine+lamivudine with nevirapine or efavirenz, according to physician choice and local standard-of-care. Children initiated abacavir+lamivudine or zidovudine+lamivudine, with nevirapine or efavirenz, following WHO dose recommendations (Supplementary Table 1).

When randomised to raltegravir, adults received 400mg raltegravir twice-daily. Adolescents aged 12-18 years and children aged 6-11 years weighing ≥25kg received the standard adult dose of raltegravir (400mg film-coated tablet twice-daily). Children 5-11 years could also receive 6 mg/kg twice-daily of a chewable tablet which could be divided into equal halves. The chewable tablet and the film-coated tablet formulations are not bioequivalent. Children weighing 10 to <14kg received 0.5 chewable tablets am and 1 pm; 14 to <20kg 1 chewable tablets am and pm; 20 to <28kg 1.5 chewable tablets am and pm; 28 to <40kg 2 chewable tablets am and pm; and 40+kg 3 chewable tablets am and pm.

(e) Further details of enhanced prophylaxis randomisation

Enhanced-prophylaxis comprised single-dose albendazole (400mg), 5-days azithromycin (500mg once-daily), 12-weeks fluconazole (100mg once-daily), and 12-weeks of a fixed-dose-combination (FDC) of co-trimoxazole (800/160mg)/isoniazid (300mg)/pyridoxine (25mg) as a scored once-daily tablet. Doses were halved for children 5-<13 years (except albendazole). After 12 weeks, fluconazole was stopped and either co-trimoxazole or the FDC continued in the enhanced-prophylaxis group; co-trimoxazole was continued or switched to the FDC in the standard-prophylaxis group. Isoniazid/pyridoxine use beyond 12 weeks depended on national IPT guidelines. Screening for active tuberculosis disease before randomisation used a WHO-based symptom checklist, with sputum examination and chest X-ray where possible. Participants already receiving or needing antimicrobial treatment or prophylaxis pragmatically received it outside the randomised design, and received other prophylaxis according to randomisation. Furthermore, any patient who became eligible for immediate isoniazid prophylaxis after trial recruitment (for example, if another household contact was diagnosed with tuberculosis) started it immediately.

Following national guidelines, all children received routine de-worming at 24 and 48 weeks post-enrolment irrespective of randomisation.

(f) Further details of endpoint ascertainment

SAEs were defined following the International Committee for Harmonization as events which led to death, were life-threatening, caused or prolonged hospitalisation (excluding elective procedures), caused permanent disability, or were other medical conditions with a real, not hypothetical risk of one of the previous categories.

HIV-1 RNA VL was assayed blinded to randomisation using the Roche COBAS Ampliprep/Taqman v2.0 in Uganda (Joint Clinical Research Centre (JCRC)), the NucliSENS EasyQ HIV-1 v2.0 in Zimbabwe (UZ-CRC, samples run at the Flow Cytometry Centre, Harare) and the Abbott m2000sp/rt 0.6mls protocol in Kenya (samples from AMPATH, Eldoret and KEMRI, Kilifi) and Blantyre (samples run at John Hopkins Research Project Laboratory, Malawi), with lower limit of detection of 20-50 copies/ml.

(g) qBody circumference and skinfold thicknesses
In one site (Harare), participants were additionally assessed at trial enrolment, weeks 12 and 48 for two body-circumferences (waist, hip) and four skinfold thicknesses using Holtain calipers (triceps, subscapular, supra-iliac, and mid-thigh). Body circumferences and skinfold thicknesses were assessed following a standardised manual of operations in triplicate.

(h) Sample size calculation from the trial protocol
1800 adults and children provided at least 80% power to detect a 50% relative reduction in 24 week all-cause mortality from 7% to 3.5%, or a 60% relative reduction from a lower mortality of 5% to 2% (two-sided alpha=0.05) allowing 5% lost to follow-up by 24 weeks, and incorporating a single inflation factor[1] to allow for the factorial design (rates in multiple groups should be lower than that in any single group) and 5% lost to follow-up at 48 weeks. The sample size calculation assumed that at least one of the three interventions tested would be ineffective and not therefore impact sample size, and therefore only a single inflation factor was used. For 90% power, the detectable reductions were 7% to 3% and 6% to 2.2% respectively. If ~10% of patients were already receiving isoniazid/fluconazole prophylaxis or ready-to-use therapeutic food at randomisation, the study design retained >80% power to detect slightly larger reductions from 7% to 3% (57% reduction). Randomizing 400 children provided at least 80% power to detect a 0.29 greater absolute increase in weight-for-age from 0 to 24 weeks with any intervention, based on other assumptions above.

(i) Statistical methods
Time-to-event analyses measured time from randomisation, censored at the last clinical follow-up if the outcome had not occurred. The primary analyses stratified for randomisation stratification factors (stratified logrank test and stratified Cox regression); results from secondary unstratified analyses were very similar (data not shown). Lost-to-follow-up was defined as not being seen in the clinic for more than 3 months (91 days).

Analyses of causes of death, and all time-to-event outcomes which did not include all-cause mortality, used competing risks methods. These estimated the probability of the event (analogous to Kaplan-Meier) using cumulative incidence functions, and estimated the effect of randomised group on the subdistribution hazard corresponding to the cumulative incidence function[2]. These analyses were conducted unstratified, as stratification is not possible with the estimating equation approach used for estimation. All endpoints based on new disease occurrences (e.g. time to new tuberculosis disease) included events identified as cause of death by the Endpoint Review Committee if these had not already been reported as new disease events before death. To identify grade 4 AEs either definitely/probably or definitely/probably/possibly related to RUSF, the Endpoint Review Committee were asked to adjudicate a relationship to all possible study interventional drugs (RUSF, raltegravir, fluconazole, isoniazid, albendazole, azithromycin and pyridoxine) to avoid providing them with details of interventional drugs actually received which would unblind them. An unblinded clinical reviewer reviewed the grade 3/4 laboratory-only events (with no clinical event associated) for relatedness to drugs actually received. In analysis, grade 4 AEs related to RUSF were defined as those related to RUSF received within 30 days prior to the event.

CD4, weight, BMI, hemoglobin and body composition parameters were compared between randomised groups over time using generalized estimating equations (GEE) (independent correlation structure), adjusting for stratification factors and scheduled visit week as categorical independent variables. The closest measurement to each scheduled visit date within equally spaced windows was used as the measurement at each scheduled visit. Continuous measurements were modeled using change from baseline as the outcome using a normal distribution. Adherence measures were modeled as dichotomous outcomes using a logistic distribution. Baseline values were those nearest to but before and within 42 days of randomisation. For body circumferences and skinfold thicknesses, the mean of the three measurements at each visit was used, and values were also truncated at the 1st and 99th percentiles to reduce influence from outliers.

(j) Subgroup analyses
We pre-specified in the protocol subgroup analyses by the other factorial randomisations and stratification factors (age </≥13 years (analyzed as 5-17, 18-29, 30-39, 40+ years because only 40 patients <13 years), centre), and also by country, baseline CD4 (0-24, 25-49, 50-99 cells per µL), initial backbone NRTI, initial NNRTI, Tuberculin Skin Test (TST) status (positive vs negative), and BMI (adults) or weight-for-age (children) (categorized at 20 kg/m^2 or -1 respectively). TST was not performed and therefore this subgroup analysis was not done. Subgroup analyses were conducted unstratified to avoid losing information from small strata with no events in one randomised group. Additional exploratory subgroup analyses were done by sex, VL (<100,000, 100,000-999,999,

≥1,000,000 copies/ml), WHO stage, BMI categorized at 18.5 kg/m^2 (or at -1 weight-for-age in those <13 years), and whether RUTF was prescribed at randomisation (rather than RUSF). Subgroup analyses were also done by whether participants reported enough food to provide everyone in the household with regular meals, and whether participants were from households that grew food crops.

References for supplementary material

1. Babiker AG, Walker AS. Statistical issues emerging from clinical trials in HIV infection. In: Geller N, ed. *Advances in clinical trial biostatistics*: CRC Press; 2003.
2. Fine JP, Gray RJ. A Proportional Hazards Model for the Subdistribution of a Competing Risk. *Journal of the American Statistical Association.* 1999;94(446):496-509.

Supplementary Tables

(k) Supplementary Table 1: Additional baseline characteristics and ART received

**Factor**	**No-RUSF (N=908)**	**RUSF (N=897)**	**All (N=1805)**
Creatinine clearance† (ml/min) (N=1793)	97.1 (77.8-120.1)	97.3 (76.6-121.9)	97.3 (77.3-121.1)
Days from screening to enrolment (ART initiation)	5 (2-8)	5 (2-8)	5 (2-8)
Initiated ART with efavirenz	820 (90.3%)	799 (89.1%)	1619 (89.7%)
Initiated ART with tenofovir/ emtricitabine backbone	716 (78.9%)	706 (78.7%)	1422 (78.8%)
Randomised to initiate ART with raltegravir as fourth drug	454 (50.0%)	448 (49.9%)	902 (50.0%)
Randomised to receive enhanced prophylaxis	456 (50.2%)	450 (50.2%)	906 (50.2%)
At last follow-up, on first-line ART	888 (97.8%)	877 (97.8%)	1765 (97.8%)
At last follow-up, on first-line ART having made within-class substitutions	60 (6.6%)	59 (6.6%)	119 (6.6%)

† estimated using Cockcroft Gault, normalized to 1.73m^2
Note: showing n(%) or median (IQR) [range].

(l) Supplementary Table 2: Receipt of RUSF and RUTF according to baseline BMI

	**No-RUSF (N=908)**	**RUSF (N=897)**	**All (N=1805)**
Baseline BMI <16 kg/m^2	111 (100%)	108 (100%)	219 (100%)
RUSF	0	90 (83.3%)	90 (41.1%)
RUTF	27 (24.3%)	18 (16.7%)	45 (20.5%)
Neither	84 (75.7%)	0	84 (38.4%)
Baseline BMI 16-<18.5 kg/m^2			
RUSF	1 (0.4%)	247 (98.0%)	248 (48.0%)
RUTF	7 (2.6%)	5 (2.0%)	12 (2.3%)
Neither	257 (97.0%)	0	257 (49.7%)
Baseline BMI ≥18.5 kg/m^2			
RUSF	0	534 (99.8%)	534 (50.3%)
RUTF	2 (0.4%)	1 (0.2%)	3 (0.3%)
Neither	524 (99.6%)	0	524 (49.4%)

Note: 8 participants had missing baseline BMI; 2 of the 6 in the no-RUSF group were prescribed RUTF.

(m) Supplementary Table 3: Causes of death through 48 weeks

	**No-RUSF (N=908)**	**RUSF (N=897)**	**All (N=1805)**
Not known to have died	794 (87.4%)	786 (87.6%)	1580 (87.5%)
Died from (predominant cause)			
Tuberculosis	23 (2.5%)	19 (2.1%)	42 (2.3%)
Cryptococcal disease	11 (1.2%)	6 (0.7%)	17 (0.9%)
Severe bacterial infections	15 (1.7%)	18 (2.0%)	33 (1.8%)
Other cause	24 (2.6%)	21 (2.3%)	45 (2.5%)
Cause not known	41 (4.5%)	47 (5.2%)	88 (4.9%)

(n) Supplementary Table 4: Secondary and other outcomes through 48 weeks

	No RUSF N (%) [events]	RUSF N (%) [events]	Total N (%) [events]	HR** (95% CI)	p
Death	114 (12.6%) [114]	111 (12.4%) [111]	225 (12.5%) [225]	0.98 (0.75,1.27)	0.98
New WHO 4 event or death	160 (17.6%) [214]	159 (17.7%) [205]	319 (17.7%) [419]	1.00 (0.80, 1.25)	0.99
New WHO 3 or 4 event or death	203 (22.4%) [305]	200 (22.3%) [306]	403 (22.3%) [611]	1.02 (0.84, 1.24)	0.86
New tuberculosis disease*	79 (8.7%) [102]	77 (8.6%) [94]	156 (8.6%) [196]	0.98 (0.72, 1.35)	0.91
New cryptococcal disease*	16 (1.8%) [28]	16 (1.8%) [23]	32 (1.8%) [51]	1.02 (0.51, 2.04)	0.96
New candida disease*	16 (1.8%) [17]	17 (1.9%) [19]	33 (1.8%) [36]	1.08 (0.54, 2.14)	0.83
New presumptive severe bacterial infection*	41 (4.5%) [55]	34 (3.8%) [56]	75 (4.2%) [111]	0.84 (0.53, 1.33)	0.45
IRIS	83 (9.1%) [86]	92 (10.3%) [94]	175 (9.7%) [180]	1.13 (0.84, 1.52)	0.43
Any serious adverse event	205 (22.6%) 254	205 (22.9%) 284	410 (22.7%) [538]	1.02 (0.84, 1.23)	0.81
New hospitalisation*	171 (18.8%) [209]	169 (18.8%) [222]	340 (18.8%) [431]	1.00 (0.81, 1.24)	0.99
Grade 4 AE*	172 (18.9%) [238]	181 (20.2%) [266]	353 (19.6%) [504]	1.07 (0.87, 1.32)	0.45
Grade 3 or 4 AE	331 (36.5%) [507]	327 (36.5%) [546]	658 (36.5%) [1053]	1.01 (0.87, 1.18)	0.90
Grade 4 AE definitely, probably or possibly related to RUSF	-	8 (0.9%) [8]	8 (0.4%) [8]	-	-
Grade 4 AE definitely or probably related to RUSF	-	0 (0.0%) [0]	0 (0.0%) [0]	-	-
AE leading to RUSF discontinuation*	-	46 (5.1%) [51]		-	-

* secondary outcome
** subhazard ratio for outcomes not including death as a component of the composite
Note: Table shows number of patients with one or more episode (% of patients) [number of episodes]
(e.g., '2 (20.0%) [3],' would indicate a total of 3 episodes in 2 patients). No evidence of interaction with other factorial randomisations (p>0.1; 30 tests).

(o) Supplementary Table 5: Serious adverse events

	No-RUSF N=908	RUSF N=897	Total N=1805	p
**Body system**				
**Any**	**205 (22.6%) 254**	**205 (22.9%) 284**	**410 (22.7%) 538**	**0.91**
**CNS**	**23 (2.5%) 23**	**26 (2.9%) 28**	**49 (2.7%) 51**	**0.67**
Acute altered conscious level	1	0	1	
Acute focal neurological event without fever, Cryptococcal disease serum CRAG +ve only	0	1	1	
Disorientated/confusion	2	1	3	
Dizziness	0	1	1	
Encephalitis – presumed infectious	0	2	2	
Encephalopathy – unspecified	1	0	1	
Encephalopathy – unspecified, Stroke, cerebrovascular accident	1	0	1	
Epilepsy, fits, convulsions	0	1	1	
Headache	1	1	2	
Hemiparesis	0	2	2	
Inter-cranial pressure	1	0	1	
Meningitis - other	0	1	1	
Meningitis lumber puncture diagnosed – no organism (no culture), HIV associated nephropathy, anemia with no clinical symptoms	0	1	1	
Meningitis lumber puncture diagnosed – no organism (no culture), Pneumonia no organism identified, Candidiasis of esophagus, trachea, bronchi or lungs, Neutropenia	1	0	1	
Meningitis no lumber puncture	0	1	1	
Myelopathy	1	0	1	
PML	0	2	2	
Peripheral neuropathy - motor only, Metabolic disorder - other	1	0	1	
Peripheral neuropathy - sensory & motor	1	0	1	
Pyogenic meningitis - no organism	0	2	2	
Pyogenic meningitis - organism	1	0	1	
Stroke, cerebrovascular accident	3	2	5	
Stroke, cerebrovascular accident, Cardiomyopathy, Tuberculosis - disseminated/miliary	1	0	1	
Stroke, cerebrovascular accident, Salmonella bacteremia, Non-typhi	0	1	1	
Toxoplasmosis of the brain	1	1	2	
Other CNS disease	0	2	2	
Brain syndrome/indeterminate intracerebral lesions	5	3	8	

	No-RUSF N=908	RUSF N=897	Total N=1805	p
Brain syndrome/indeterminate intracerebral lesions, Anemia with clinical symptoms	0	1	1	
Brain syndrome/indeterminate intracerebral lesions, Anemia with clinical symptoms, Hypoglycemia, Raised creatinine	0	1	1	
Brain syndrome/indeterminate intracerebral lesions, Presumed septicemia/bacteremia - not investigated, Lung syndrome, Renal failure - acute, Anemia with clinical symptoms	0	1	1	
Brain syndrome/indeterminate intracerebral lesions, Tuberculosis - pulmonary - smear positive	1	0	1	
**Psychiatric**	**6 (0.7%) 6**	**6 (0.7%) 6**	**12 (0.7%) 12**	**1.00**
Depression	1	0	1	
Psychosis, mania	5	4	9	
Psychosis, mania, Neutropenia	0	1	1	
Psychosis, mania, Tuberculosis - pulmonary - smear positive, Pneumonia no organism identified	0	1	1	
**Lower Respiratory Tract**	**17 (1.9%) 18**	**12 (1.3%) 15**	**29 (1.6%) 33**	**0.45**
Candidiasis of esophagus, trachea, bronchi or lungs	1	1	2	
Chest infection	2	0	2	
Pleural effusion - other, Pneumonia no organism identified	1	0	1	
Pneumonia - Pneumocystis carinii (PCP)	1	0	1	
Pneumonia - Pneumocystis carinii (PCP), Anemia with no clinical symptoms	1	0	1	
Pneumonia - other bacterial	0	1	1	
Pneumonia - other bacterial, Anemia with clinical symptoms	0	1	1	
Pneumonia no organism identified	10	7	17	
Pneumonia no organism identified, Ascites, Hepatitis B	0	1	1	
Pneumonia no organism identified, Hypotension/shock/toxic shock	0	1	1	
Pulmonary embolism, Deep vein thrombosis	1	2	3	
Lung syndrome, Anemia with clinical symptoms	1	1	2	
**Cardiovascular**	**5 (0.6%) 6**	**8 (0.9%) 10**	**13 (0.7%) 16**	**0.42**
Chest pain	1	0	1	
Cardiomyopathy, Tuberculosis - disseminated/miliary	1	0	1	
Congestive cardiac failure, Cardiomyopathy, Anemia with clinical symptoms	1	0	1	
Congestive cardiac failure, HIV Associated Cardiomyopathy, Other cardiovascular, Deep vein thrombosis	0	1	1	
Congestive cardiac failure, Pancytopenia, bone marrow depression	0	1	1	
Deep vein thrombosis	2	4	6	
Deep vein thrombosis, Anemia with clinical symptoms	0	2	2	
Deep vein thrombosis, Candidiasis of esophagus, trachea, bronchi or lungs, Gastroenteritis	0	1	1	
Deep vein thrombosis, Lymphadenopathy	0	1	1	

	No-RUSF N=908	RUSF N=897	Total N=1805	p
Deep vein thrombosis, Pleural effusion - Tuberculosis	1	0	1	
**Gastrointestinal**	**13 (1.4%) 16**	**19 (2.1%) 19**	**32 (1.8%) 35**	**0.29**
Abdominal or epigastric pain	0	1	1	
Acute abdomen, Tuberculosis - abdominal	0	1	1	
Appendicitis	1	0	1	
Dysphagia, difficulty swallowing	0	2	2	
Gastroenteritis	5	7	12	
Gastroenteritis, Hepatitis cause unknown	1	0	1	
Gastroenteritis, Raised creatinine	0	1	1	
Gastroenteritis, Renal failure - acute	0	1	1	
Hematemesis	0	1	1	
Indigestion, esophageal reflux, gastritis, ulcerative esophagitis	2	2	4	
Pancreatitis	2	0	2	
Per rectal bleeding (fresh blood and/or malaena)	0	1	1	
Vomiting	4	2	6	
Vomiting, Abdominal or epigastric pain	1	0	1	
**Diarrheal**	**5 (0.6%) 5**	**2 (0.2%) 2**	**7 (0.4%) 7**	**0.45**
Acute diarrhea not investigated	3	0	3	
Chronic diarrhea not investigated, Oral candida	0	1	1	
Chronic diarrhea not investigated, Renal failure - acute	1	0	1	
Chronic diarrhea not investigated, Septicemia with organism (unspecified), Pancytopenia, bone marrow depression	0	1	1	
Chronic diarrhea with cryptosporidia	1	0	1	
**Wasting Syndrome**	**1 (0.1%) 1**	**1 (0.1%) 1**	**2 (0.1%) 2**	**1.00**
Severe weight loss (>10%)	0	1	1	
Severe weight loss (>10%), Anemia with clinical symptoms, Raised creatinine	1	0	1	
**Hepatic**	**5 (0.6%) 6**	**8 (0.9%) 8**	**13 (0.7%) 14**	**0.42**
Acute hepatitis	3	4	7	
Acute hepatitis, Candidiasis of esophagus, trachea, bronchi or lungs, Anemia with clinical symptoms	1	0	1	
Hepatic encephalopathy, Hepatic failure - chronic, Tuberculosis - disseminated/miliary, Hepatitis B	0	1	1	
Hepatic failure - acute	0	1	1	
Hepatic failure - acute, Tuberculosis - pulmonary - smear negative or not done, Wasting syndrome uninvestigated	2	0	2	
Hepatic failure - acute, Tuberculosis - pulmonary - smear positive, Renal failure - acute, Hepatitis B, Neutropenia	0	1	1	

	No-RUSF N=908	RUSF N=897	Total N=1805	p
Jaundice	0	1	1	
**Renal**	**11 (1.2%) 11**	**9 (1.0%) 10**	**20 (1.1%) 21**	**0.82**
HIV associated nephropathy	0	1	1	
HIV associated nephropathy, Tuberculosis - pulmonary - smear positive	0	1	1	
Hematuria, Anemia with clinical symptoms	0	1	1	
Pyelonephritis	1	2	3	
Pyelonephritis, Renal failure - acute	1	0	1	
Renal failure - acute	3	1	4	
Renal failure - acute, Acute diarrhea not investigated, Intravascular hemolysis	0	1	1	
Renal failure - acute, Acute diarrhea not investigated, Oral candida	0	2	2	
Renal failure - acute, Anemia with clinical symptoms	1	0	1	
Renal failure - acute, Anemia with clinical symptoms, Tuberculosis - lymph nodes, Kaposi's sarcoma lymph nodes, Overdose (not suicide attempt), Peripheral neuropathy - sensory & motor	1	0	1	
Renal failure - acute, Gastroenteritis	1	0	1	
Renal failure - acute, Gastroenteritis, Anemia with clinical symptoms	0	1	1	
Renal failure - acute, Pyelonephritis	1	0	1	
Renal failure - acute, Raised AST, Raised ALT	1	0	1	
Renal failure - acute, Tuberculosis - other, Oral candida	1	0	1	
**Genitourinary**	**0 (0.0%) 0**	**3 (0.3%) 3**	**3 (0.2%) 3**	**0.12**
Lower urinary tract infection (UTI), cystitis, Neutropenia	0	1	1	
Lower urinary tract infection (UTI), cystitis, Raised creatinine, Hypertension	0	1	1	
Vaginal bleeding	0	1	1	
**Musculoskeletal**	**0 (0.0%) 0**	**1 (0.1%) 1**	**1 (0.1%) 1**	**0.50**
Pyomyositis - infection	0	1	1	
**Skin**	**1 (0.1%) 1**	**2 (0.2%) 2**	**3 (0.2%) 3**	**0.62**
Hypersensitivity reaction	0	2	2	
Hypersensitivity reaction, Epilepsy, fits, convulsions	1	0	1	
**Hematological**	**15 (1.7%) 16**	**17 (1.9%) 19**	**32 (1.8%) 35**	**0.72**
Anemia with clinical symptoms	10	13	23	
Anemia with clinical symptoms, Gastroenteritis	0	1	1	
Anemia with clinical symptoms, Gastroenteritis, Neutropenia	1	0	1	
Anemia with clinical symptoms, Headache	1	0	1	
Anemia with clinical symptoms, Hemiparesis	0	1	1	

	No-RUSF N=908	RUSF N=897	Total N=1805	p
Anemia with clinical symptoms, Neutropenia	1	1	2	
Anemia with clinical symptoms, Neutropenia, Tuberculosis - pulmonary - smear positive	0	1	1	
Anemia with clinical symptoms, Renal failure - acute	1	0	1	
Anemia with clinical symptoms, Skin abscess, Neutropenia	0	1	1	
Anemia with clinical symptoms, Thrombocytopenia	0	1	1	
Anemia with no clinical symptoms, Neutropenia, Thrombocytopenia	1	0	1	
Pancytopenia, bone marrow depression	1	0	1	
**Biochemical**	**1 (0.1%) 1**	**1 (0.1%) 1**	**2 (0.1%) 2**	**1.00**
Metabolic disorder - other, Candidiasis of esophagus, trachea, bronchi or lungs	1	0	1	
Raised bilirubin, Raised liver enzymes	0	1	1	
**Systemic**	**6 (0.7%) 6**	**7 (0.8%) 8**	**13 (0.7%) 14**	**0.79**
Wasting syndrome uninvestigated	3	2	5	
Wasting syndrome uninvestigated, Abdominal or epigastric pain	0	1	1	
Wasting syndrome uninvestigated, Acute hepatitis	1	0	1	
Wasting syndrome uninvestigated, Gastroenteritis	0	1	1	
Wasting syndrome uninvestigated, Oral candida	1	0	1	
Stevens-Johnson Syndrome	0	2	2	
Stevens-Johnson Syndrome, Presumed septicemia/bacteremia - not investigated	0	1	1	
Dehydration, Tuberculosis - abdominal	0	1	1	
Dehydration, Vomiting	1	0	1	
**Specific Infections**	**87 (9.6%) 100**	**90 (10.0%) 105**	**177 (9.8%) 205**	**0.75**
Cryptococcal meningitis	14	17	31	
Cryptococcal meningitis, Anemia with no clinical symptoms	1	0	1	
Cryptococcal meningitis, Cirrhosis, candidiasis of esophagus, trachea, bronchi or lungs	1	0	1	
Cryptococcal meningitis, Raised ALT	1	0	1	
Cryptococcal meningitis, Renal failure - acute	0	1	1	
Cryptococcal meningitis, Renal failure - acute, Anemia with clinical symptoms	0	1	1	
Cryptococcal meningitis, Tuberculosis - disseminated/miliary	1	0	1	
Tuberculosis - meningitis	1	1	2	
Tuberculosis - meningitis, Candidiasis of esophagus, trachea, bronchi or lungs	1	0	1	
Tuberculosis - meningitis, Pneumonia no organism identified, Oral candida	1	0	1	
Pleural effusion - Tuberculosis	1	0	1	
Pleural effusion - Tuberculosis, P falciparum malaria	1	0	1	

	No-RUSF N=908	RUSF N=897	Total N=1805	p
Tuberculosis - pulmonary - smear negative or not done	4	2	6	
Tuberculosis - pulmonary - smear negative or not done, Aspiration pneumonia	0	1	1	
Tuberculosis - pulmonary - smear negative or not done, Hepatitis cause unknown	1	0	1	
Tuberculosis - pulmonary - smear negative or not done, Neutropenia	0	1	1	
Tuberculosis - pulmonary - smear negative or not done, Pneumonia - other bacterial	0	1	1	
Tuberculosis - pulmonary - smear negative or not done, Severe malnutrition, Pneumonia - other bacterial, Anemia with clinical symptoms	1	0	1	
Tuberculosis - pulmonary - smear positive	5	9	14	
Tuberculosis - pulmonary - smear positive, Anemia with clinical symptoms	0	2	2	
Tuberculosis - pulmonary - smear positive, Oral candida	0	2	2	
Tuberculosis - pulmonary - smear positive, Pneumonia no organism identified	1	0	1	
Tuberculosis - pulmonary - smear positive, Renal failure - acute, Anemia with clinical symptoms, Hyponatremia	0	1	1	
Tuberculosis - pulmonary - smear positive, Tuberculosis - lymph nodes	0	1	1	
CMV retinitis	1	1	2	
Tuberculosis - abdominal	1	5	6	
Tuberculosis - abdominal, Anemia with no clinical symptoms, Acute hepatitis	0	1	1	
Tuberculosis - abdominal, Peripheral neuropathy - sensory & motor, Ulcer, decubitus ulcer	0	1	1	
Cutaneous warts, Human Papillomavirus	0	1	1	
Tuberculosis - lymph nodes	4	2	6	
Tuberculosis - lymph nodes, Acute hepatitis, Rash, urticaria	1	0	1	
Tuberculosis - lymph nodes, Anemia with clinical symptoms	1	0	1	
Tuberculosis - lymph nodes, Jaundice	1	0	1	
Tuberculosis - lymph nodes, Renal failure - acute	0	1	1	
Mycobacterial disease - atypical disseminated	0	1	1	
Tuberculosis - disseminated/miliary	13	15	28	
Tuberculosis - disseminated/miliary, Acute hepatitis	1	1	2	
Tuberculosis - disseminated/miliary, Anemia with clinical symptoms	2	1	3	
Tuberculosis - disseminated/miliary, Anemia with clinical symptoms, Presumed septicemia/bacteremia - no organism, Renal failure - acute	0	1	1	
Tuberculosis - disseminated/miliary, Ascites, Pleural effusion - other, P falciparum malaria	1	0	1	
Tuberculosis - disseminated/miliary, Candidiasis of esophagus, trachea, bronchi or lungs	0	1	1	
Tuberculosis - disseminated/miliary, Chronic diarrhea not investigated, Acute hepatitis	0	1	1	
Tuberculosis - disseminated/miliary, Hepatic failure - acute, Anemia with clinical symptoms	1	0	1	
Tuberculosis - disseminated/miliary, Hepatic failure - acute, Renal failure - acute, Candidiasis of esophagus,	1	0	1	

	No-RUSF N=908	RUSF N=897	Total N=1805	p
trachea, bronchi or lungs				
Tuberculosis - disseminated/miliary, Kaposi's sarcoma cutaneous	1	0	1	
Tuberculosis - disseminated/miliary, Neutropenia	0	1	1	
Tuberculosis - disseminated/miliary, Oral candida	0	1	1	
Tuberculosis - disseminated/miliary, Pneumonia - other bacterial	0	1	1	
Tuberculosis - disseminated/miliary, Pneumonia no organism identified	1	0	1	
Tuberculosis - disseminated/miliary, Pneumonia no organism identified, Anemia with no clinical symptoms, Visceral abscess	0	1	1	
Tuberculosis - disseminated/miliary, Pneumonia no organism identified, Jaundice	0	1	1	
Tuberculosis - disseminated/miliary, Presumed septicemia/bacteremia - no organism, Hypokalemia, Purpura, bruising, petechiae, Thrombocytopenia, Low albumin	1	0	1	
Tuberculosis - disseminated/miliary, Presumed septicemia/bacteremia - not investigated	0	3	3	
Tuberculosis - disseminated/miliary, Presumed septicemia/bacteremia - not investigated, Anemia with clinical symptoms	1	0	1	
Tuberculosis - disseminated/miliary, Presumed septicemia/bacteremia - not investigated, Pancytopenia, bone marrow depression	0	1	1	
Tuberculosis - disseminated/miliary, Pure red cell aplasia	5	0	5	
Tuberculosis - disseminated/miliary, Raised liver enzymes	0	3	3	
Tuberculosis - disseminated/miliary, Renal failure - acute	2	0	2	
Tuberculosis - disseminated/miliary, Thrombocytopenia	1	0	1	
Tuberculosis - other	1	0	1	
Tuberculosis - other, Candidiasis of esophagus, trachea, bronchi or lungs, Hypophosphatasemia	0	1	1	
Cryptococcal fungemia	0	1	1	
Presumed septicemia/bacteremia - no organism	5	0	5	
Presumed septicemia/bacteremia - no organism, Anemia with clinical symptoms	1	0	1	
Presumed septicemia/bacteremia - no organism, Anemia with clinical symptoms, P falciparum malaria	0	1	1	
Presumed septicemia/bacteremia - no organism, Candidiasis of esophagus, trachea, bronchi or lungs	0	1	1	
Presumed septicemia/bacteremia - no organism, Disorientated/confusion, Hypoglycemia	1	0	1	
Presumed septicemia/bacteremia - no organism, Renal failure - acute, Pancytopenia, bone marrow depression	0	1	1	
Presumed septicemia/bacteremia - no organism, Renal failure - chronic	1	0	1	
Presumed septicemia/bacteremia - no organism, Visceral abscess, Candidiasis of esophagus, trachea, bronchi or lungs	0	1	1	
Presumed septicemia/bacteremia - not investigated	5	1	6	
Presumed septicemia/bacteremia - not investigated, Anemia with clinical symptoms	0	2	2	
Presumed septicemia/bacteremia - not investigated, Anemia with clinical symptoms, Neutropenia	1	0	1	

	No-RUSF N=908	RUSF N=897	Total N=1805	p
Presumed septicemia/bacteremia - not investigated, Brain syndrome/indeterminate intracerebral lesions	1	0	1	
Presumed septicemia/bacteremia - not investigated, Diabetes - Type II, Disorientated/confusion	0	1	1	
Presumed septicemia/bacteremia - not investigated, Lower urinary tract infection (UTI), cystitis	1	0	1	
Presumed septicemia/bacteremia - not investigated, Pancytopenia, bone marrow depression	1	1	2	
Presumed septicemia/bacteremia - not investigated, Pneumonia no organism identified	1	0	1	
Presumed septicemia/bacteremia - not investigated, Renal failure - acute, Anemia with clinical symptoms, Thrombocytopenia	0	1	1	
Presumed septicemia/bacteremia - not investigated, Tuberculosis - abdominal	0	1	1	
Presumed septicemia/bacteremia - not investigated, Tuberculosis - disseminated/miliary	1	0	1	
Presumed septicemia/bacteremia - not investigated, Tuberculosis - pulmonary - smear positive	0	1	1	
Presumed septicemia/bacteremia - not investigated, Wasting syndrome with investigations	0	1	1	
P falciparum malaria	3	3	6	
P falciparum malaria, Anemia with no clinical symptoms, Neutropenia	0	1	1	
P falciparum malaria, Neutropenia	2	0	2	
Histoplasmosis, Anemia with clinical symptoms	0	1	1	
**Tumors**	**9 (1.0%) 9**	**13 (1.4%) 16**	**22 (1.2%) 25**	**0.40**
Primary CNS lymphoma	1	0	1	
Kaposi's sarcoma pulmonary	0	1	1	
Kaposi's sarcoma mucosal	1	0	1	
Kaposi's sarcoma cutaneous	0	7	7	
Kaposi's sarcoma cutaneous, Anemia with clinical symptoms	1	1	2	
Kaposi's sarcoma lymph nodes	1	1	2	
Kaposi's sarcoma lymph nodes, Nephrotic syndrome	1	0	1	
Non Hodgkin's lymphoma	0	1	1	
Benign tumor	1	0	1	
Other Solid tumor	1	2	3	
Kaposi's sarcoma - other	0	2	2	
Kaposi's sarcoma - other, Acute hepatitis	0	1	1	
Kaposi's sarcoma - other, Anemia with clinical symptoms	1	0	1	
Kaposi's sarcoma - other, Presumed septicemia/bacteremia - not investigated	1	0	1	
**Abscess**	**2 (0.2%) 2**	**1 (0.1%) 1**	**3 (0.2%) 3**	**1.00**
CNS abscess	1	0	1	
CNS abscess, Pyogenic meningitis - no organism	0	1	1	

	No-RUSF N=908	RUSF N=897	Total N=1805	p
Skin abscess	1	0	1	
**Non-HIV Related Deaths**	**0 (0.0%) 0**	**1 (0.1%) 1**	**1 (0.1%) 1**	**0.50**
Traumatic	0	1	1	
**Other**	**27 (3.0%) 27**	**27 (3.0%) 27**	**54 (3.0%) 54**	**1.00**
Non-fatal trauma	1	2	3	
Death, cause unknown	26	25	51	
**Unknown**	**0 (0.0%) 0**	**1 (0.1%) 1**	**1 (0.1%) 1**	
Unknown Reason for Hospitalisation	0	1	1	

Note: Table shows number of patients with one or more episode (% of patients) [number of episodes]
(e.g., '2 (20.0%) [3],' would indicate a total of 3 episodes in 2 patients)

(p) Supplementary Table 6: Adverse events and poor acceptability leading to discontinuation of RUSF

	**RUSF N=897**
**Discontinued RUSF**	**57 (6.4%)**
**Due to an adverse event (any grade)**	**46 (5.1%)**
nausea/vomiting	30 (3.3%)
painful swallowing	1 (0.1%)
nausea/vomiting, abdominal discomfort	1 (0.1%)
mouth ulcers/wounds	1 (0.1%)
abdominal discomfort/pain	4 (0.4%)
diarrhea	2 (0.2%)
generalized erythema	1 (0.1%)
renal failure	1 (0.1%)
urinary tract infection	1 (0.1%)
TB, renal failure, anemia	1 (0.1%)
hypersensitivity reaction	2 (0.2%)
confusion, oral ulcers, diarrhea	1 (0.1%)
**Due to dislike**	**11 (1.2%)**
poor tolerance/acceptability	6 (0.7%)
dislikes taste	5 (0.6%)

Supplementary Figures

(q) Supplementary Figure 1: RUSF and RUTF received over time

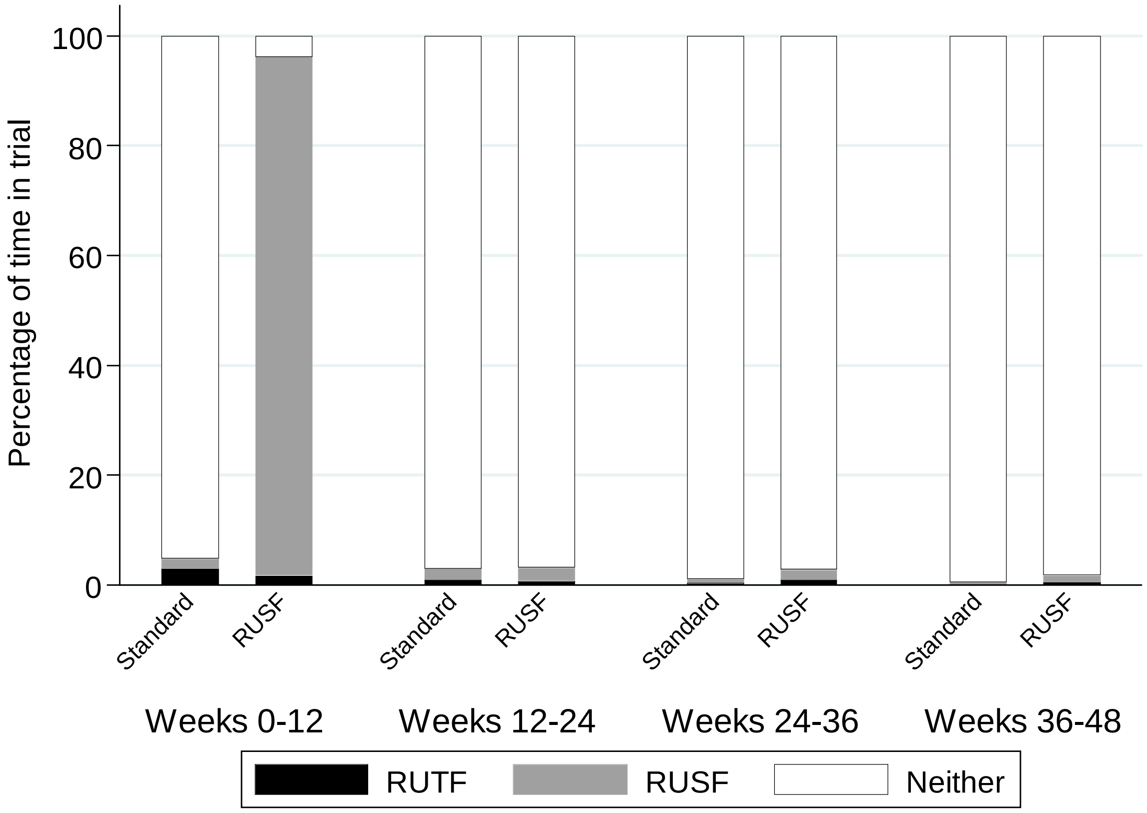

(r) Supplementary Figure 2: Self-reported adherence to RUSF and ART, and acceptability

(a) Percentage reporting missing RUSF doses in the last 4 weeks (RUSF group only)

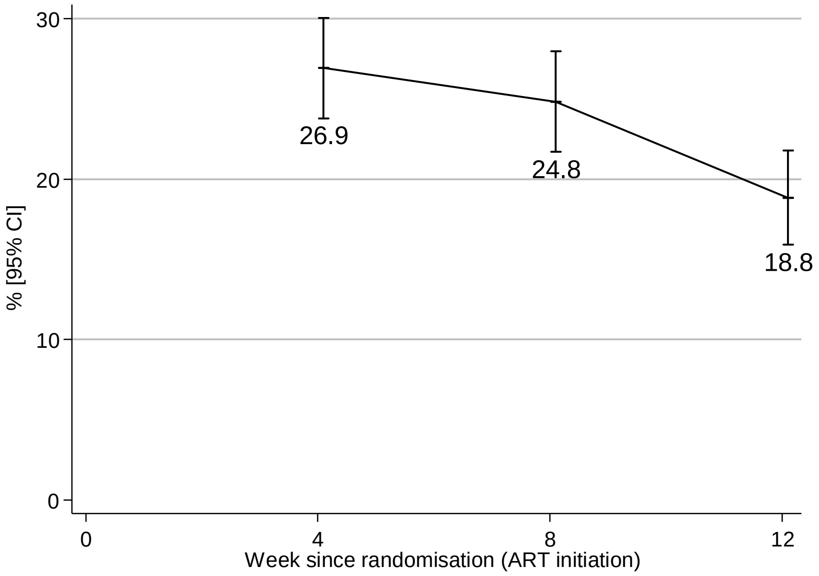

(b) RUSF acceptability at 4 and 12 weeks (RUSF group only)

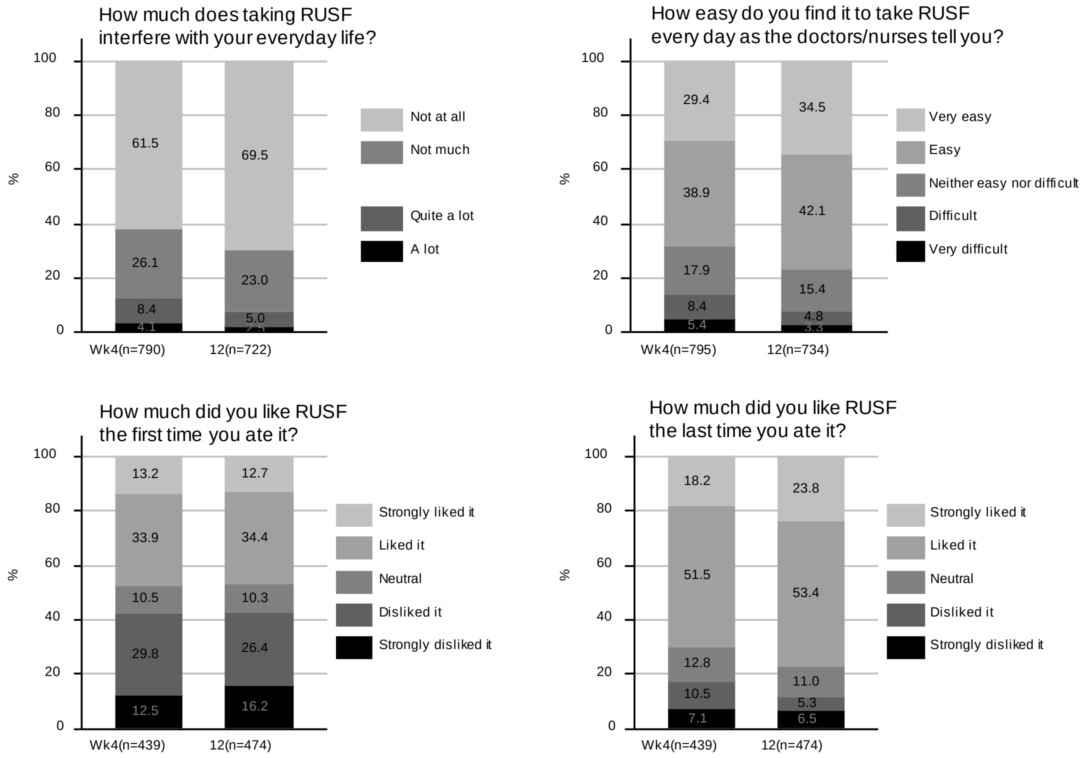

(c) ART adherence in RUSF and no RUSF groups: percentage reporting missing ART doses in the last 4 weeks

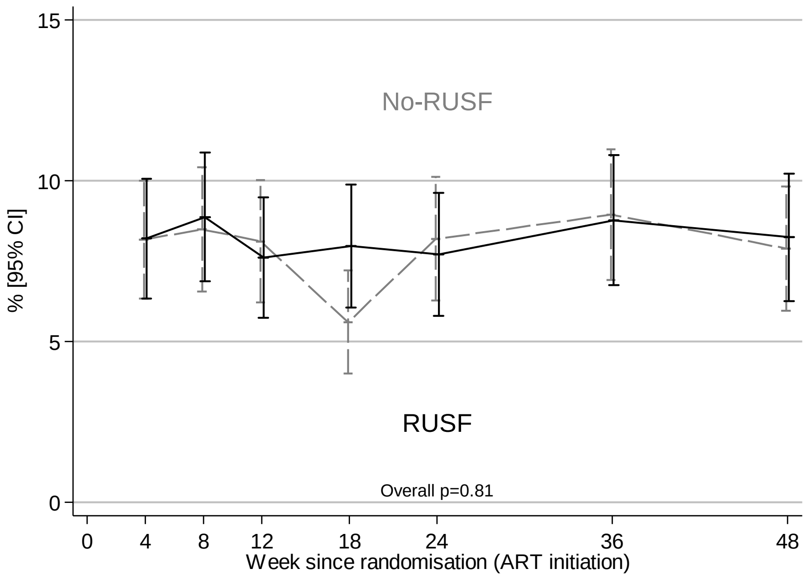

`

Percentage							
RUSF	8.2%	8.9%	7.6%	8.0%	7.7%	8.8%	8.2%
No-RUSF	8.2%	8.5%	8.1%	5.6%	8.2%	8.9%	7.9%
p	*0.99*	*0.83*	*0.67*	*0.07*	*0.65*	*0.68*	*0.90*

(s) **Supplementary Figure 3: Subgroup analyses for primary endpoint (24-week mortality)**

	No-RUSF	RUSF	HR [95% CI]	Interaction p
RANDOMIZED TO INITIATE ART WITH RALTEGRAVIR AS FOURTH DRUG				
No	45/454(9.9%)	46/449(10.2%)	1.03 [0.68, 1.56]	0.85
Yes	47/454(10.4%)	50/448(11.2%)	1.09 [0.73, 1.63]	
RANDOMIZED TO RECEIVE ENHANCED PROPHYLAXIS				
No	52/452(11.5%)	56/447(12.5%)	1.11 [0.76, 1.62]	0.75
Yes	40/456(8.8%)	40/450(8.9%)	1.01 [0.65, 1.56]	
COUNTRY				
Kenya	12/178(6.7%)	27/173(15.6%)	2.41 [1.22, 4.75]	0.04
Malawi	9/128(7.0%)	10/127(7.9%)	1.13 [0.46, 2.78]	
Uganda	51/315(16.2%)	40/315(12.7%)	0.78 [0.52, 1.18]	
Zimbabwe	20/287(7.0%)	19/282(6.7%)	0.96 [0.51, 1.80]	
CENTRE				
Blantyre	9/128(7.0%)	10/127(7.9%)	1.13 [0.46, 2.78]	0.02
Harare	20/287(7.0%)	19/282(6.7%)	0.96 [0.51, 1.80]	
Kilifi	6/73(8.2%)	20/70(28.6%)	3.91 [1.57, 9.73]	
Eldoret	6/105(5.7%)	7/103(6.8%)	1.17 [0.39, 3.48]	
Fort Portal	12/71(16.9%)	14/70(20.0%)	1.26 [0.58, 2.72]	
Mbale	10/59(16.9%)	11/60(18.3%)	1.09 [0.46, 2.57]	
Mbarara	15/116(12.9%)	6/114(5.3%)	0.40 [0.15, 1.02]	
Gulu	14/69(20.3%)	9/71(12.7%)	0.59 [0.26, 1.37]	
AGE AT LAST BIRTHDAY (YEARS)				
5-17	2/41(4.9%)	1/31(3.2%)	0.64 [0.06, 7.11]	0.60
18-29	23/202(11.4%)	19/211(9.0%)	0.79 [0.43, 1.45]	
30-39	27/353(7.6%)	36/358(10.1%)	1.33 [0.81, 2.19]	
>=40	40/312(12.8%)	40/297(13.5%)	1.06 [0.68, 1.64]	
CD4 (CELLS/MM^3)				
0-<25	52/344(15.1%)	44/312(14.1%)	0.92 [0.62, 1.38]	0.31
25-<50	23/258(8.9%)	23/251(9.2%)	1.04 [0.58, 1.85]	
>=50	17/306(5.6%)	29/334(8.7%)	1.60 [0.88, 2.92]	
INITIAL BACKBONE NRTI				
3TC ABC	3/30(10.0%)	7/30(23.3%)	2.49 [0.64, 9.64]	0.30
3TC TDF or FTC TDF	68/716(9.5%)	72/706(10.2%)	1.08 [0.77, 1.50]	
3TC ZDV	21/162(13.0%)	17/161(10.6%)	0.82 [0.43, 1.56]	
INITIAL NNRTI				
EFV	81/820(9.9%)	88/799(11.0%)	1.12 [0.83, 1.52]	0.27
NVP	11/88(12.5%)	8/98(8.2%)	0.65 [0.26, 1.62]	
BMI (KG/M^2) (WEIGHT-FOR-AGE IF <13 YEARS)				
>= 20 (w-f-a >= -1)	17/358(4.7%)	22/346(6.4%)	1.36 [0.72, 2.56]	0.45
< 20 (w-f-a < -1)	70/545(12.8%)	73/549(13.3%)	1.04 [0.75, 1.44]	
SEX*				
Male	46/487(9.4%)	45/474(9.5%)	1.00 [0.66, 1.51]	0.70
Female	46/421(10.9%)	51/423(12.1%)	1.12 [0.75, 1.67]	
HIV VIRAL LOAD (C/ML)*				
<100000	18/233(7.7%)	21/237(8.9%)	1.15 [0.61, 2.15]	0.91
100000-999999	53/530(10.0%)	54/506(10.7%)	1.08 [0.74, 1.57]	
>=1000000	21/145(14.5%)	21/153(13.7%)	0.95 [0.52, 1.75]	
WHO STAGE*				
1	11/152(7.2%)	7/148(4.7%)	0.66 [0.25, 1.69]	0.68
2	17/293(5.8%)	19/261(7.3%)	1.28 [0.66, 2.46]	
3	42/338(12.4%)	43/353(12.2%)	0.97 [0.64, 1.49]	
4	22/125(17.6%)	27/135(20.0%)	1.15 [0.65, 2.02]	
BMI (KG/M^2) (WEIGHT-FOR-AGE IF <13 YEARS)*				
>= 18.5 (w-f-a >= -1)	34/528(6.4%)	43/538(8.0%)	1.26 [0.80, 1.98]	0.50
< 18.5 (w-f-a < -1)	53/375(14.1%)	52/357(14.6%)	1.03 [0.70, 1.51]	
RUTF PRESCRIBED AT RANDOMISATION*				
No	82/870(9.4%)	91/873(10.4%)	1.11 [0.83, 1.50]	0.56
Yes	10/38(26.3%)	5/24(20.8%)	0.83 [0.28, 2.43]	
HOUSEHOLD FOOD SUFFICIENT TO FEED*				
No	33/271(12.2%)	32/280(11.4%)	0.93 [0.57, 1.51]	0.51
Yes	57/627(9.1%)	62/610(10.2%)	1.13 [0.79, 1.62]	
GROW OWN CROPS*				
No	43/408(10.5%)	37/413(9.0%)	0.85 [0.55, 1.31]	0.20
Yes	47/489(9.6%)	56/475(11.8%)	1.24 [0.84, 1.83]	

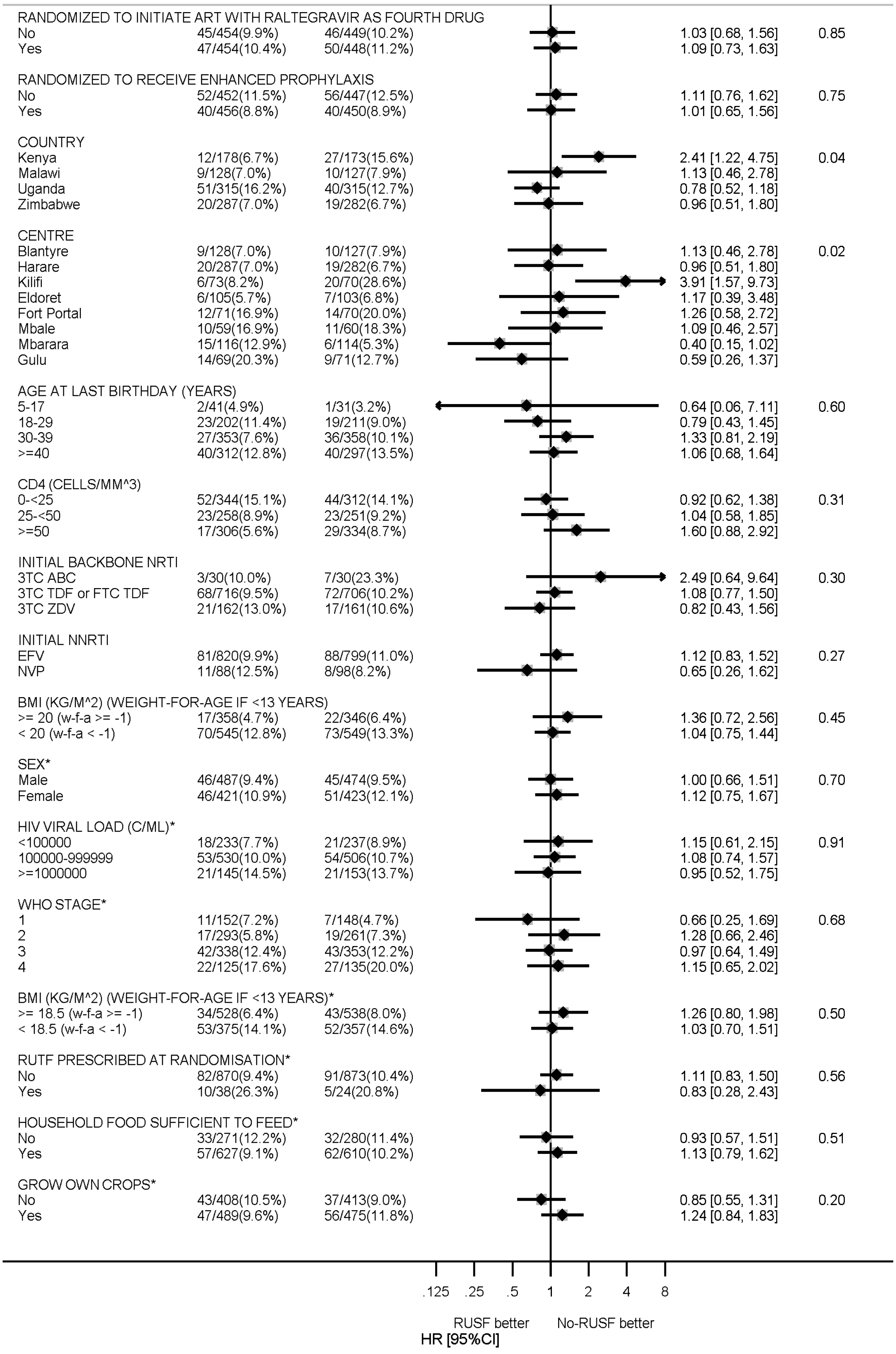

* exploratory analysis

(t) Supplementary Figure 4: HIV viral load, CD4 cell count and ART adherence

(a) HIV viral load <50 copies/ml

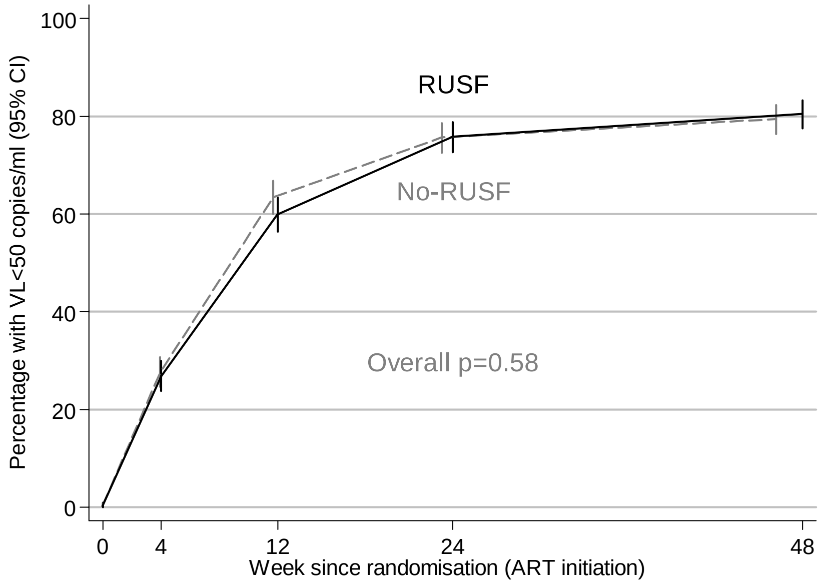

Percentage				
RUSF	26.8%	59.9%	75.8%	80.5%
No-RUSF	27.6%	63.4%	75.7%	79.4%
p	*0.71*	*0.15*	*0.95*	*0.61*

(b) CD4

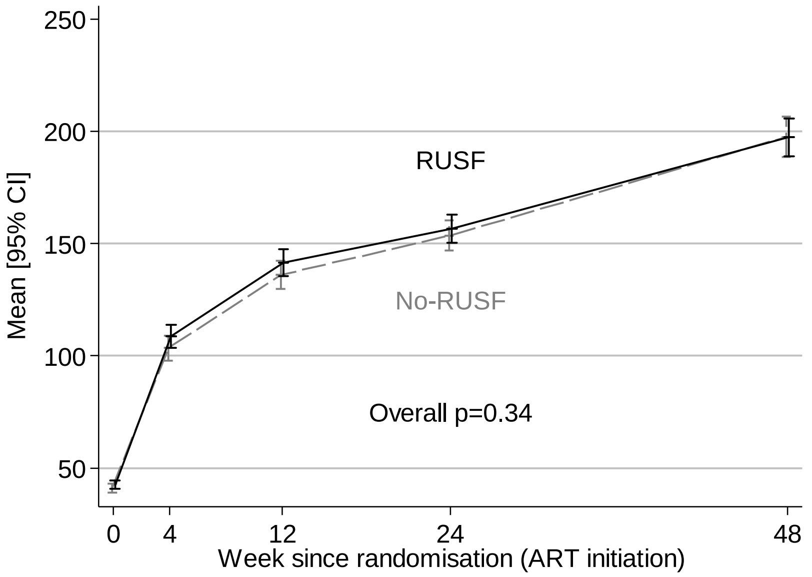

Change from baseline				
RUSF	+66	+98	+113	+154
No-RUSF	+62	+94	+111	+155
p	*0.20*	*0.22*	*0.46*	*0.87*

Note: p-value for (b) compares change from baseline across randomised groups

(u) Supplementary Figure 5: (a) Mean BMI and (b) changes in BMI (kg/m^2) according to BMI at ART initiation

(a) Mean BMI

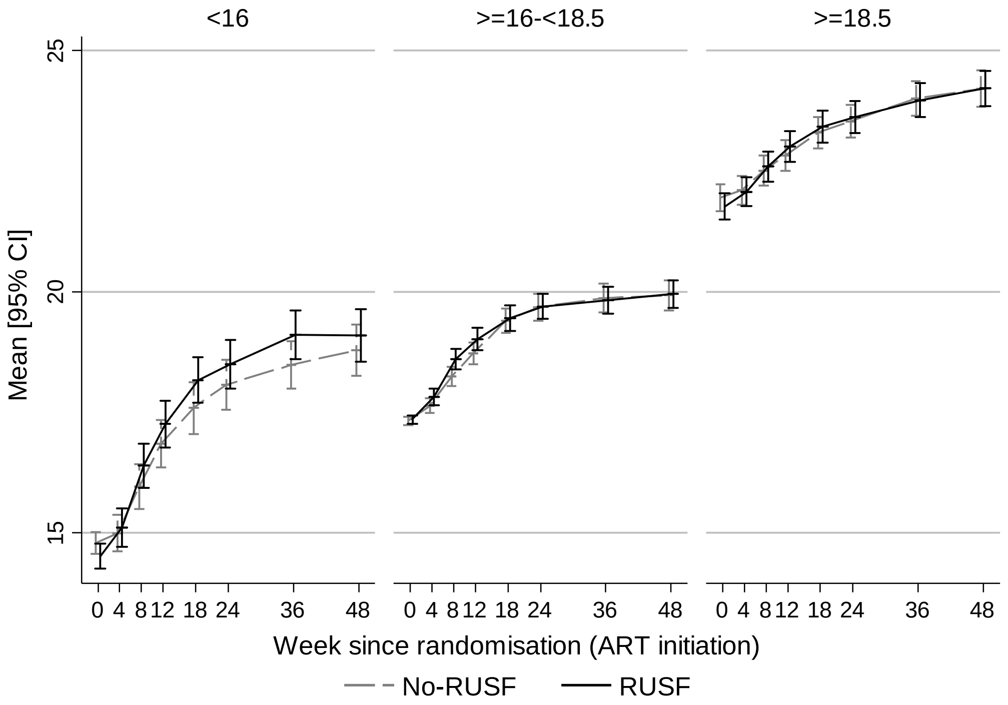

(b) Mean change in BMI

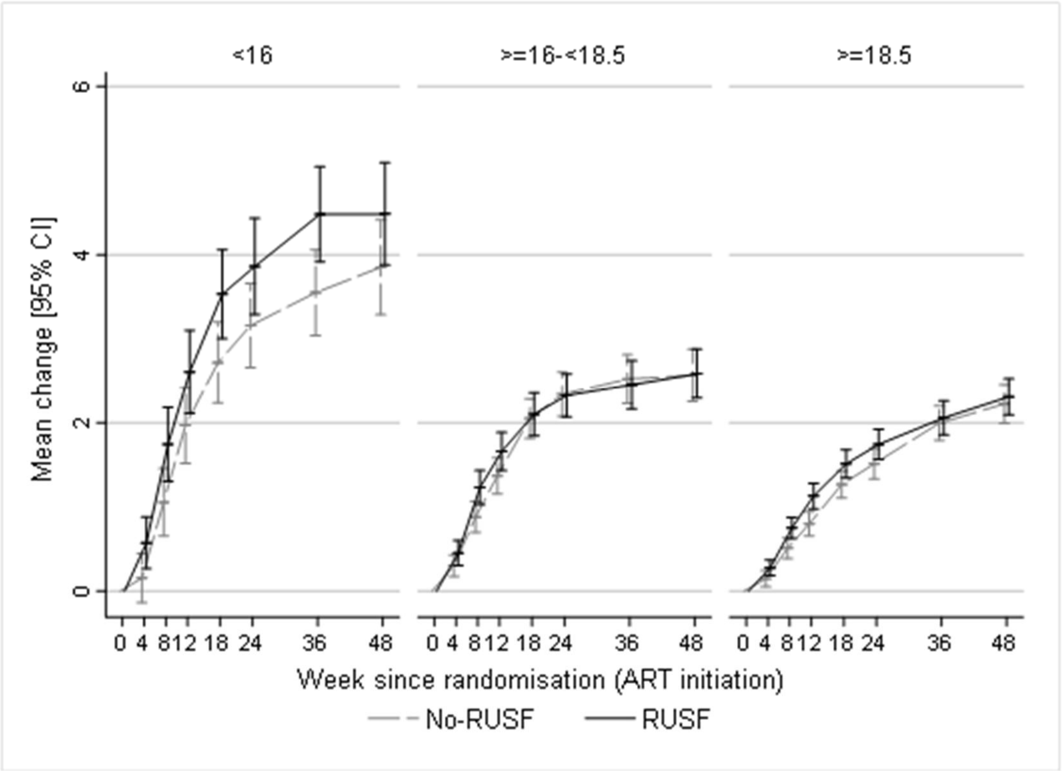

Note:

Note: at 12 weeks mean change in baseline in RUSF vs no-RUSF was +2.6 vs +2.0 kg for those <16 kg/m^2, +1.7 vs +1.4 kg for those 16-<18.5 kg/m^2 and +1.1 vs +0.8 kg for those ≥18.5 kg/m^2 at baseline. $p_{heterogeneity}$=0.80 comparing change from baseline between randomised groups across baseline BMI subgroups at week 12.

(v) Supplementary Figure 6: Changes in body-circumferences and skinfold thicknesses (participants from Harare only)

(a) Triceps skinfold (mm)

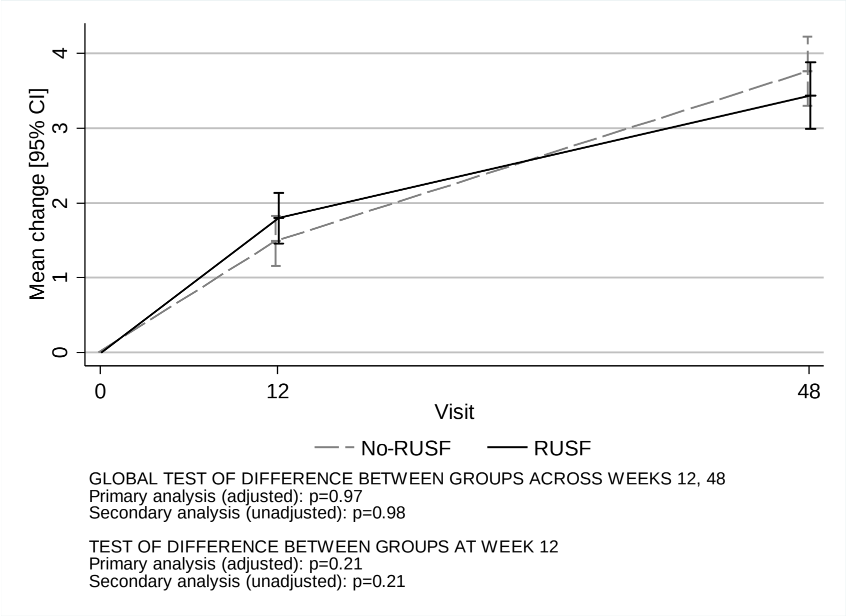

(b) Subscapular skinfold (mm)

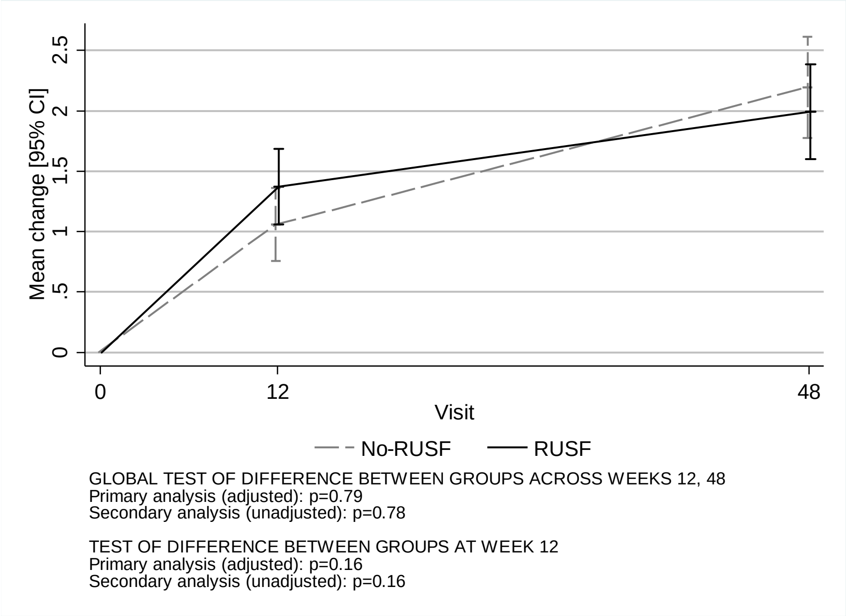

(c) Supra-iliac skinfold (mm)

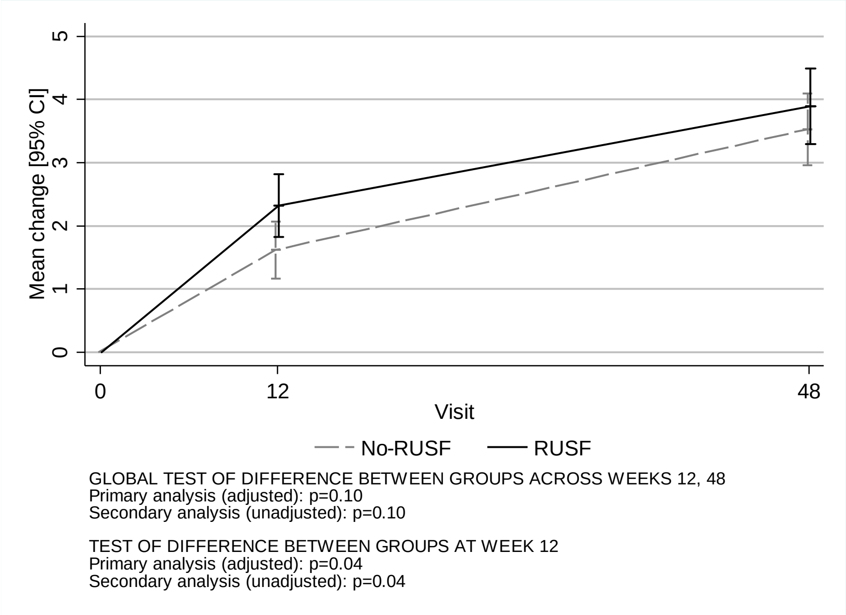

(d) Mid-thigh skinfold (mm)

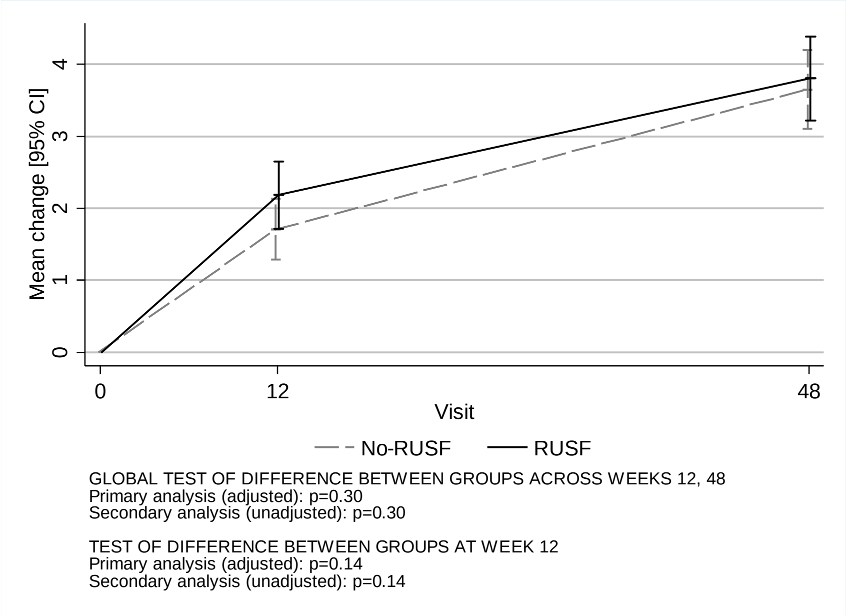

(e) Sum of four skinfolds (mm)

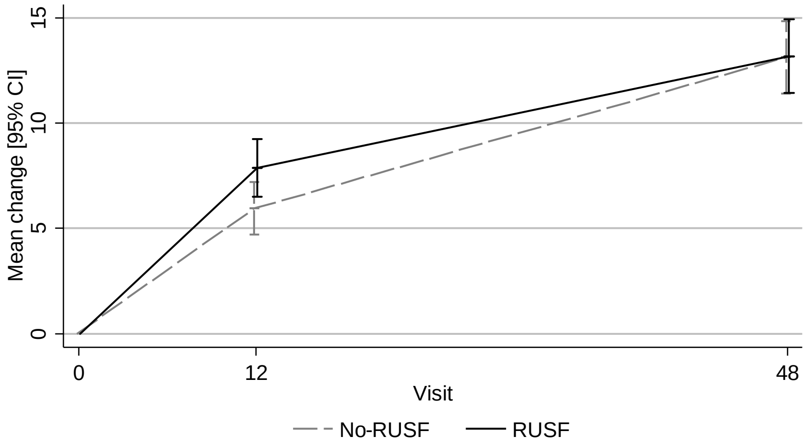

GLOBAL TEST OF DIFFERENCE BETWEEN GROUPS ACROSS WEEKS 12, 48
Primary analysis (adjusted): p=0.28
Secondary analysis (unadjusted): p=0.28

TEST OF DIFFERENCE BETWEEN GROUPS AT WEEK 12
Primary analysis (adjusted): p=0.04
Secondary analysis (unadjusted): p=0.04

(f) Waist circumference (cm)

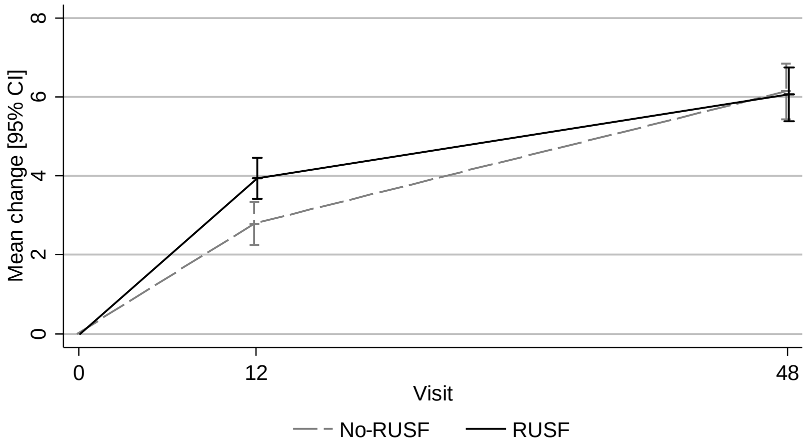

GLOBAL TEST OF DIFFERENCE BETWEEN GROUPS ACROSS WEEKS 12, 48
Primary analysis (adjusted): p=0.14
Secondary analysis (unadjusted): p=0.14

TEST OF DIFFERENCE BETWEEN GROUPS AT WEEK 12
Primary analysis (adjusted): p=0.003
Secondary analysis (unadjusted): p=0.003

(g) Hip circumference (cm)

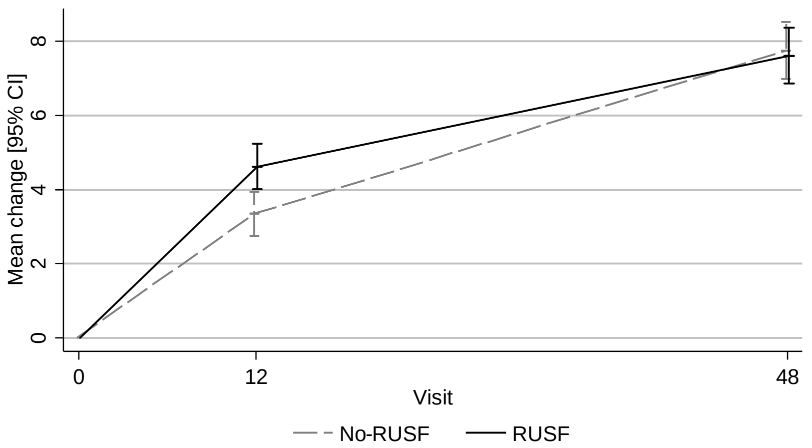

GLOBAL TEST OF DIFFERENCE BETWEEN GROUPS ACROSS WEEKS 12, 48
Primary analysis (adjusted): p=0.17
Secondary analysis (unadjusted): p=0.17

TEST OF DIFFERENCE BETWEEN GROUPS AT WEEK 12
Primary analysis (adjusted): p=0.003
Secondary analysis (unadjusted): p=0.003

(h) Waist-hip ratio

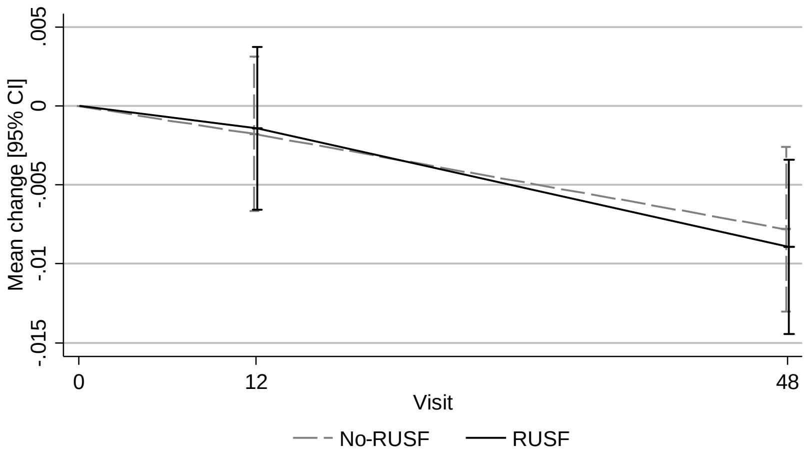

GLOBAL TEST OF DIFFERENCE BETWEEN GROUPS ACROSS WEEKS 12, 48
Primary analysis (adjusted): p=0.90
Secondary analysis (unadjusted): p=0.91

TEST OF DIFFERENCE BETWEEN GROUPS AT WEEK 12
Primary analysis (adjusted): p=0.92
Secondary analysis (unadjusted): p=0.92

(w) Supplementary Figure 7: Weight and BMI-for-age Z score changes for participants aged <13 years (N=40; 23 no-RUSF, 17 RUSF)

(a) Change in weight-for-age Z score

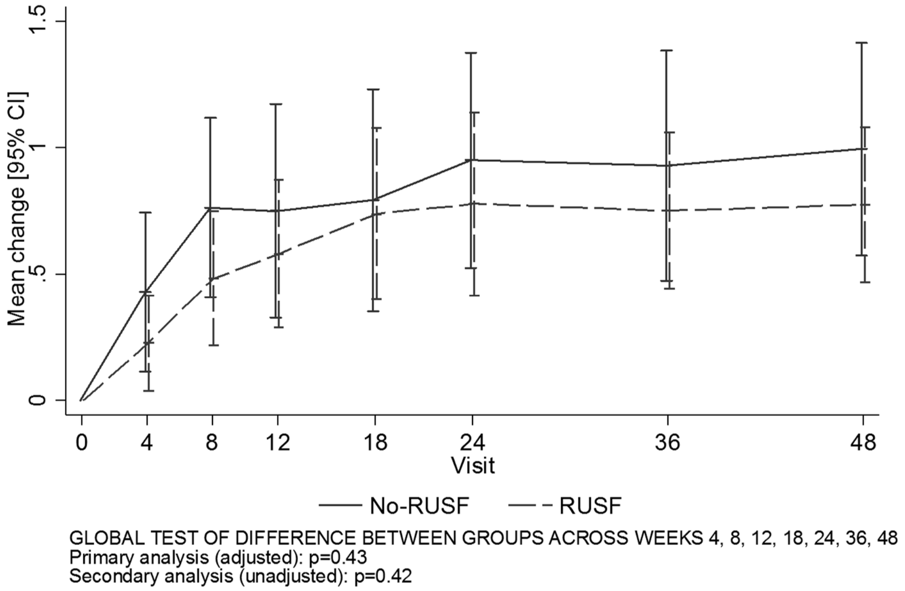

(b) Change in BMI-for-age Z score

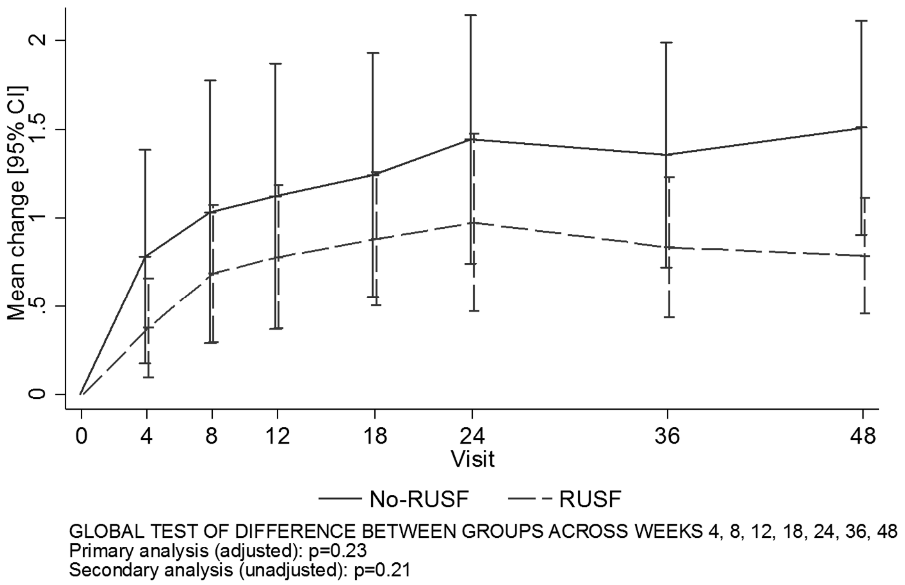

Supplement: Supplementary appendix [file mmc1.pdf]
